# Single-cell sequencing reveals homogeneity and heterogeneity of the cytopathological mechanisms in different etiology-induced AKI

**DOI:** 10.1038/s41419-023-05830-z

**Published:** 2023-05-11

**Authors:** Zhimin Chen, Yinshuang Li, Ying Yuan, Kunmei Lai, Keng Ye, Yujiao Lin, Ruilong Lan, Hong Chen, Yanfang Xu

**Affiliations:** 1 Department of Nephrology, Blood Purification Research Center, the First Affiliated Hospital, Fujian Medical University, Fuzhou, 350005 China; 2 Research Center for Metabolic Chronic Kidney Disease, the First Affiliated Hospital, Fujian Medical University, Fuzhou, 350005 China; 3 Department of Nephrology, National Regional Medical Center, Binhai Campus of the First Affiliated Hospital, Fujian Medical University, Fuzhou, 350212 China; 4 Central laboratory, the First Affiliated Hospital, Fujian Medical University, Fuzhou, 350005 China; 5 Department of Pathology, the First Affiliated Hospital, Fujian Medical University, Fuzhou, 350005 China

**Keywords:** Transcriptomics, Cell death

## Abstract

Homogeneity and heterogeneity of the cytopathological mechanisms in different etiology-induced acute kidney injury (AKI) are poorly understood. Here, we performed single-cell sequencing (scRNA) on mouse kidneys with five common AKI etiologies (CP-Cisplatin, IRI-Ischemia-reperfusion injury, UUO-Unilateral ureteral obstruction, FA-Folic acid, and SO-Sodium oxalate). We constructed a potent multi-model AKI scRNA atlas containing 20 celltypes with 80,689 high-quality cells. The data suggest that compared to IRI and CP-AKI, FA- and SO-AKI exhibit injury characteristics more similar to UUO-AKI, which may due to tiny crystal-induced intrarenal obstruction. Through scRNA atlas, 7 different functional proximal tubular cell (PTC) subtypes were identified, we found that Maladaptive PTCs and classical *Havcr1* PTCs but not novel *Krt20* PTCs affect the pro-inflammatory and pro-fibrotic levels in different AKI models. And cell death and cytoskeletal remodeling events are widespread patterns of injury in PTCs. Moreover, we found that programmed cell death predominated in PTCs, whereas apoptosis and autophagy prevailed in the remaining renal tubules. We also identified *S100a6* as a novel AKI-endothelial injury biomarker. Furthermore, we revealed that the dynamic and active immune (especially *Arg1* Macro_2 cells) -parenchymal cell interactions are important features of AKI. Taken together, our study provides a potent resource for understanding the pathogenesis of AKI and early intervention in AKI progression at single-cell resolution.

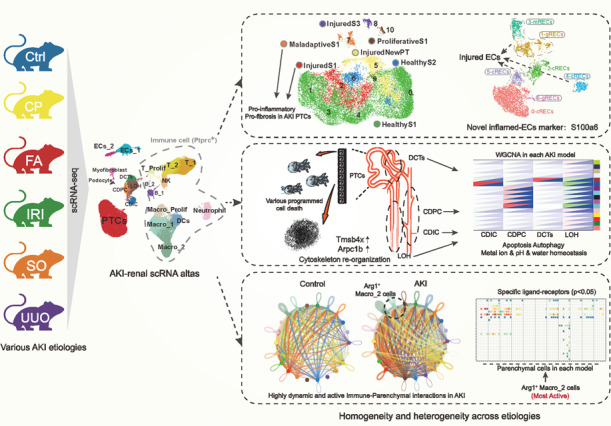

## Introduction

Acute kidney injury (AKI) is a complex systemic disease and a common complication in critically ill patients [1, 2]. Current studies suggest that AKI increases the risk of chronic kidney disease (CKD) by nearly 8–10-fold, making it a primary risk factor for CKD pathogenesis [3, 4]. Serum creatinine (Scr) is the most commonly used index for assessing renal function, but the observed changes in Scr are lagging [5, 6]. Moreover, Scr levels are not useful as a functional index to distinguish parenchymal versus functional impairment and they are susceptible to factors such as weight, circulating volume, and medications [7, 8]. Therefore, as well as elucidating the injury mechanisms at critical sites of AKI kidney, there is also an urgent need to identify biomarkers of early injury at different segments to help prevent kidney injury, and halt progression to CKD [9].

In recent years, rapid advances in single-cell sequencing technologies have provided unprecedented resolution for studying disease homo- and heterogeneity [10–12]. For example, Kirita et al. [13] identified distinct pro-inflammatory and pro-fibrotic proximal tubular cells (FR-PTCs) using single nuclear RNA sequencing (snRNA-seq) kidneys of ischemia reperfusion injury (IRI) -AKI mice. Rudman-Melnick et al. [14] found that AKI dramatically altered ligand-receptor crosstalk in kidney cells by using snRNA-seq of kidneys at different time points in unilateral IRI-AKI mice. Balzer et al. [15] found that renal proximal tubule cells are critically vulnerable cells in IRI models, and they identified a specific cluster of maladaptive/fibrotic proximal tubules after prolonged ischemia that expresses pro-inflammatory and pro-fibrotic cytokines and myeloid chemokines. However, due to the renal pathological features and mechanisms of AKI induced by various etiologies (such as drugs and crystals) are different [16, 17], a comprehensive understanding of the molecular mechanisms behind different etiologies induced AKI is imperatively needed, which will provide precise treatment strategies and novel biomarkers for predicting AKI progression. Furthermore, compared to single-cell RNA sequencing (scRNA-seq), snRNA-seq did not provide comprehensive and well resolve data regarding the impact of immune cell infiltration on renal parenchymal cells during AKI [18–20].

In this study, we established five common mouse models of kidney injury and made the different etiologies comparable by controlling the modeling time and performing single-cell sequencing at the early stages of kidney injury within the AKI time frame. The five models successfully replicated the four common AKI etiologies: cisplatin (for drug)-induced kidney injury (CP), IRI for pre-renal AKI, unilateral ureteral obstruction (UUO) for obstructive AKI, and folic acid (FA), sodium oxalate (SO) for crystalloid AKI [21–26]. By using the high-resolution perspective of single-cell transcriptomics, we constructed a richly detailed single-cell atlas of AKI containing 9 renal parenchymal cell types and 11 immune cell subtypes. We aimed to comprehensively compare the similarities and differences in immune cell composition, parenchymal cell proportions, and Intercellular ligand-receptor patterns induced by different etiologies of AKI during similar stages of kidney injury. Additionally, we aimed to explore the characteristics of endothelial cells and different segmental renal tubular cells in injury, as well as the dominant regulatory cell death patterns in the critical PTCs of AKI injury, and identify new biological markers of PTC injury. Through these aspects to reveal the potential model heterogeneity and injury homogeneity among different etiologically induced AKI.

## Results

### Multi-model AKI-scRNA atlas with 20 cell types reveals differences in immune, parenchymal cell proportions between different models

**Fig. 1:**
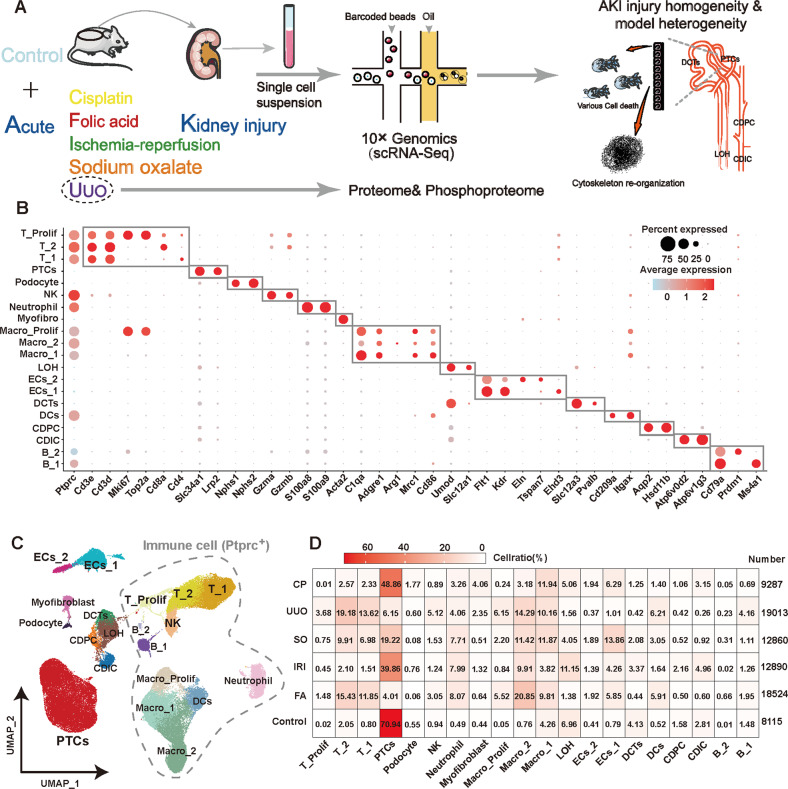
The construction of the scRNA atlas initially explored the potential homogeneity and heterogeneity among different etiology-induced AKI. **A** Study overview. Renal single-cell transcriptome sequencing was performed on control and five common mouse models of AKI: cisplatin kidney (CP), folic acid (FA), sodium oxalate (SO), ischemia reperfusion injury (IRI), and unilateral ureteral obstruction (UUO). Additionally, proteome and phosphorylated proteome was performed on UUO mouse kidneys to collectively reveal the potential homo- and heterogeneity of injury patterns in different etiology-induced AKI. **B**, **C** Data from 6 groups were integrated as a single dataset after data quality control and removal of batch effects. A total of 33 clusters were identified by unsupervised clustering, combined with top differentially expressed genes for each cluster and known cell markers for renal cell types, we identified a total of 20 cell subtypes, including 11 *Ptptc* (CD45) immune cells and 9 renal parenchymal cells. *n* = 2 each group. **D** Heatmap of model-celltype ratios. Compared with the IRI and CP models, the cell proportions of the FA, SO model are closer to those of the UUO model. *n* = 2 each group. Abbreviations: proximal tubular cells (PTCs), loop of Henle (LOH), distal convoluted tubules (DCTs), endothelial cells (ECs), collecting duct-principal cells (CDPC), collecting duct-intercalated cells (CDIC), dendritic cells (DCs), natural killer cells (NK), myofibroblasts (Myofibro), macrophage (Macro).

We performed scRNA-seq of kidneys from five AKI models and Control samples (Fig. 1A). The AKI status of the mice was confirmed by analyzing the levels of Scr, blood urea nitrogen (BUN), and pathological changes in the kidneys (Supplemental Fig. S1). After the initial cellranger quality control, SoupX package were used to correct the ambient RNA contamination. Low-quality cells were excluded according to set criteria. Double cells were removed and potential batch effects were corrected by the harmony algorithm (Supplemental Fig. S2, S3). A total of 20 celltypes were annotated, with 80,689 high-quality cells, including 11 *Ptptc* (*CD45*) immune cells: T_1 cells (*Cd3Cd4*), T_2 cells (*Cd3Cd8a*), Proliferative T cells (*Cd3Mki67*), NK cells (*GzmaGzmb*), Neutrophil (*S100a8S100a9*), Macro_1 cells (M1 type macrophage, *Adgre1Cd86*), Macro_2 cells (M2 type macrophage, *Adgre1Arg1*), Proliferative Macrophages (*Adgre1Mki67*), Dendritic cells (*Cd209aItgax*), B_1 cells (Mature B cells, *Cd79aMs4a1*), and B_2 cells (Plasama cells, *Cd79aPrdm1*) and 9 renal parenchymal cells: PTCs (*Slc34a1Lrp2*), Podocyte (*Nphs1Nphs2*), Myofibroblast (*Acta2*), loop of henle (LOH, *UmodSlc12a1*), distal tuble cells (DCTs,*Slc12a3Pvalb*), collecting duct-intercalated cells (CDIC, *Atp6v0d2Atp6v1g3*), collecting duct-principal cells (CDPC, *Aqp2Hsd11b*), endothelial cells 1 (ECs_1, *Flt1Ehd3*), and endothelial cells 2 (ECs_2, *Flt1Eln*) (Fig. 1B, C; Supplemental Table S1, S2). After the onset of AKI, immune cells are heavily infiltrated and different AKI models show heterogeneity in cell ratios. More PTCs were retained in CP- and IRI-induced AKI, with the proportion of immune cells in UUO-, FA-, and SO-induced AKI being more than 50% (Fig. 1D). By flow cytometry, we confirmed the *Ptprc* (*CD45*) immune cell ratio to validate the reliability of single-cell sequencing results (Supplemental Fig. S4).

### Maladaptive PTCs and classical *Havcr1* injured PTCs but not novel *Krt20* PTCs affect the pro-inflammatory and pro-fibrotic levels in different AKI models

**Fig. 2:**
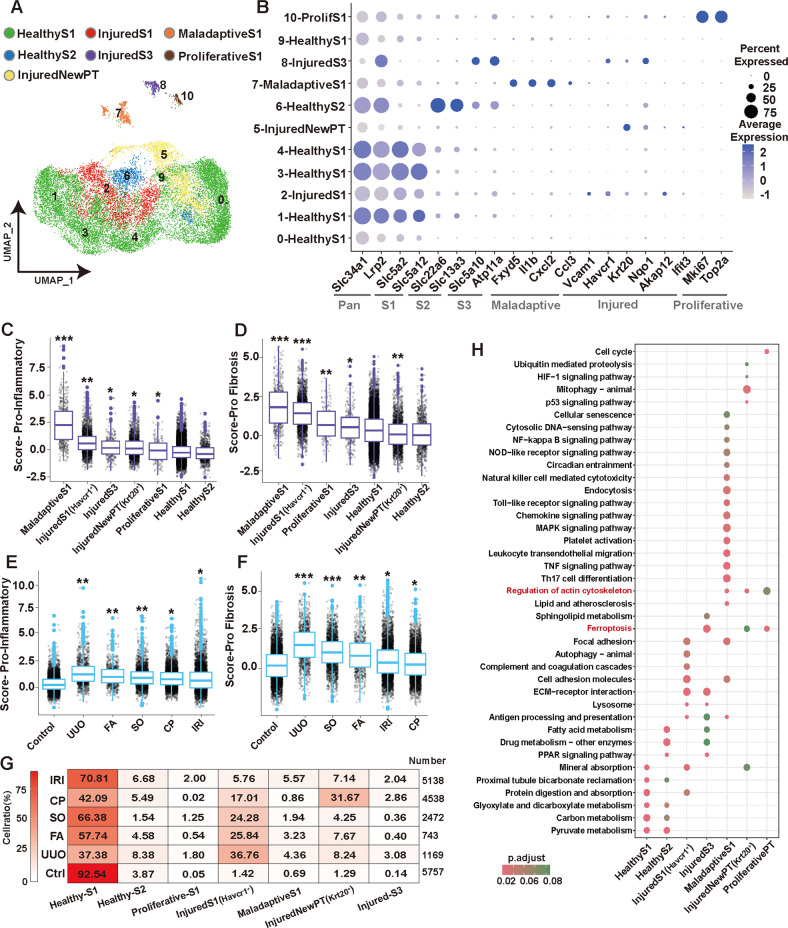
The proportion of PTCs with different injury states affects the pro-inflammatory and pro-fibrotic levels in different AKI models. **A**, **B** PTCs were isolated from the AKI dataset for re-clustering, and the Seurat standard process was repeated with batch effects removed. A total of 11 clusters were identified by known biomarkers of PTC injury and S1-, S2-, S3-segment biomarkers. A total of 7 different states of renal tubular cells were identified. *n* = 2 each group. **C**, **D** The pro-inflammation (**C**) and pro-fibrosis (**D**) levels in different status PTCs were scored using Seurat’s AddModuleScore function and sorted from left to right according to the level of the score. Wilcoxon test, *P < 0.05, **P < 0.01, ***P < 0.001 vs. Healthy-S1 group. **E**, **F** The pro-inflammation (**E**) and pro-fibrosis (**F**) levels in different etiology-induced AKI models were scored using Seurat’s AddModuleScore function and sorted from left to right according to the level of the score. Wilcoxon test, *P < 0.05, **P < 0.01, ***P < 0.001 vs. Control group. **G** The proportion of cells with 7 different states of PTCs in each model. over 95% of PTCs in Control were healthy, a large number of injured NewPTs with *Krt20* were present in CP model. While the injured PTCs in the FA, UUO and SO models were predominantly S1-Injured. Notably, the IRI model contains a large number of S1-Maladaptive PTCs. **H** Kyoto Encyclopedia of Genes and Genomes (KEGG) analysis of the molecular pathways involved in 7 different states of PTCs. Inflammation, cellular senescence, cell death, and cytoskeletal remodeling are common features of injured PTCs.

The impairment of PTCs is one of the major pathological changes in AKI [5, 27]. We re-clustered the PTCs and mapped the scores of the top three PCs to the PTC re-cluster graph. The aggregation of the AKI-specific group of PTCs is driven by variability between models within AKI (Supplemental Fig. S5A, B**)**. Further, using a series of well-known renal segmental markers and PTCs injury biomarkers [13, 15, 28, 29], we identified 7 different states of PTCs: Healthy S1 segment PTCs (*Slc34a1Slc5a2*), Healthy S2 segment PTCs (*Slc34a1Slc22a6*), InjuredS1 segment PTCs (*Slc34a1Slc5a2Havcr1*), Maladaptive S1 segment PTCs (*Slc34a1Slc5a2Fxyd5*), NewInjured PTCs (*Slc34a1Krt20*), Proliferative-PTCs (*Slc34a1Mki67*) and InjuredS3-segment PTCs (*Slc34a1Slc5a10Havcr1*). **(**Fig. 2A, B**;** Supplemental Fig. S5C, D and Supplemental Table S3). It is worth mentioned that Maladaptive PTCs is a group of injury-state PTCs identified in recent years and profoundly characterized in several recent studies [13, 15]. NewInjured PTCs (*Krt20*) are also an injury-state PTCs [30] and we verified the expression in different AKI models by immunohistochemistry (Supplemental Fig. S6). We ranked the pro-inflammatory and pro-fibrotic scores of different PTCs subtypes from highest to lowest and compared them with Healthy-S1-PTCs, respectively. We found that Maladaptive-PTCs had the highest pro-inflammatory and pro-fibrotic scores (*P* < 0.05), its high pro-inflammatory and pro-fibrotic levels suggesting that this group of cells may be the earliest group of PTCs that switch to CKD in the early stage of renal injury. Followed by InjuredS1 PTCs (*Havcr1*), while NewInjuredPT(*Krt20*) exhibited weak inflammatory and weak fibrotic properties with pro-fibrosis scores even lower than Healthy-S1 PTCs (*P* < 0.05) (Fig. 2C, D). Most of the genes upregulated in the expression profiles of Maladaptive PTCs overlap with those of macrophages, neutrophils, implying that these cells share the killing profile with immune cells (Supplemental Table S2 and S3).

We believe that the proportion of PTCs subtypes with different pro-inflammatory and pro-fibrotic capacities would affect the overall pro-inflammatory and pro-fibrotic levels of PTCs. Therefore, we compared the pro-inflammatory and pro-fibrotic scores of each model PTCs with the control group separately and arranged them from left to right according to the level of the score (Fig. 2E, F). By combining cell proportion heatmaps **(**Fig. 2G) of PTCs subtypes in different models, we found that AKI models with higher proportions of InjuredS1 (*Havcr1*) PTCs exhibited higher pro-inflammatory and pro-fibrotic scores, which was particularly significant in UUO, FA and SO models, as InjuredS1 PTCs were the major injured cell subpopulation in these models. In contrast, high proportions of NewInjuredPT (*Krt20*) seemed to have not directly contributed to the pro-inflammatory and pro-fibrotic levels of AKI-PTCs. Despite the high proportion of InjuredS1 (*Havcr1*), the high proportion of NewInjuredPT (*Krt20*) in the CP model may have weakened its pro-inflammatory and pro-fibrotic levels, resulting in levels similar to those of the IRI model. On the other hand, the IRI model had a lower proportion of both InjuredS1 and NewInjuredPT than the CP model, but its higher proportion of Maladaptive PTCs strongly enhanced the pro-inflammatory and pro-fibrotic scores (Fig. 2E–G). Lastly, by correlating the proportion of injured state PTCs cells in different models with the pro-inflammatory and pro-fibrotic scores of the models (Supplemental Fig. S7A, B), we concluded that Maladaptive PTCs and classical *Havcr1* injured PTCs but not novel *Krt20* PTCs affect the pro- inflammatory and pro-fibrotic levels in different AKI models.

### The dominant regulatory cell death mode (pyroptosis, necroptosis, and ferroptosis) differs in PTCs among different AKI models

**Fig. 3:**
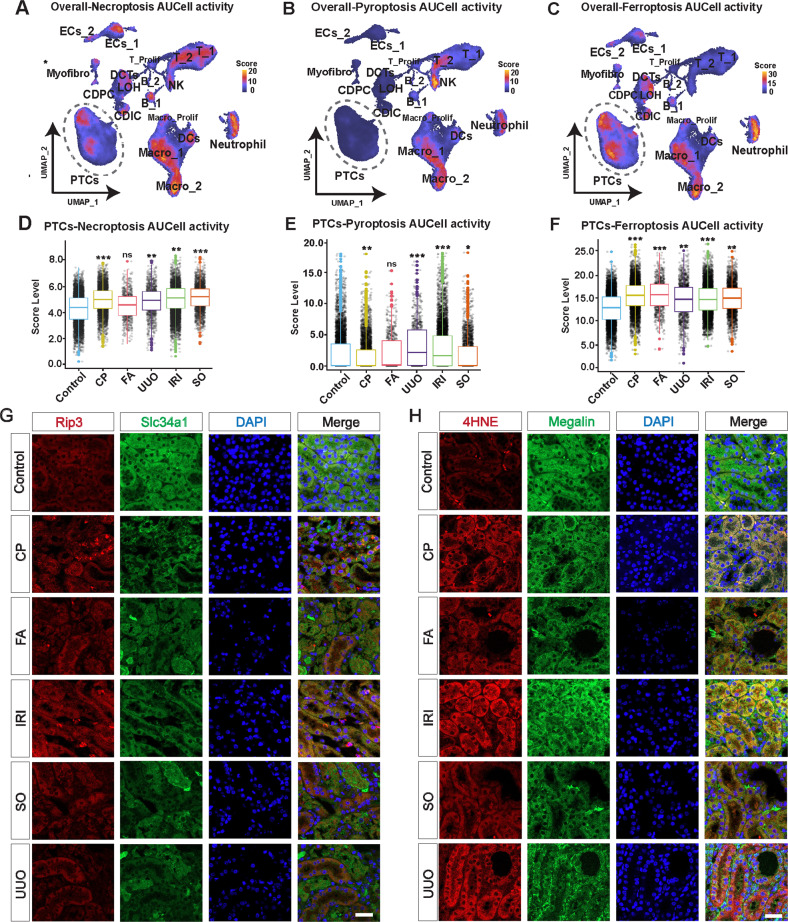
The dominant regulatory cell death mode (pyroptosis, necroptosis, and ferroptosis) differs in PTCs among different AKI models. **A**–**C** The AUCell algorithm evaluates the distribution of necroptosis (**A**), pyroptosis (**B**), and ferroptosis (**C**) pathway activity across different cell types in the overall AKI dataset, presented by a UMAP plot, with higher scoring cells being brighter in color. Quantitative analysis results are presented in Supplemental Fig S8. **D**–**F** Isolating PTCs from the overall AKI dataset for intergroup comparisons. Wilcoxon test, *P < 0.05, **P < 0.01, ***P < 0.001 vs. Control group, ns: not significant. **G**, **H** Co-staining of sections from Control, cisplatin (CP), folic acid (FA), sodium oxalate (SO), ischemia reperfusion injury (IRI), and unilateral ureteral obstruction (UUO) kidneys for Rip3 (**G**), 4HNE (**H**) (red) and Megalin or Slc34a1 (green). Scale bars = 100 μM. Quantitative analysis of protein immunofluorescence intensity in Supplemental Fig. S9A.B. Rip3 is a key molecule in necroptosis and 4HNE is one of the markers of ferroptosis. The results showed that both necroptosis and ferroptosis were involved in the injury process of PTCs.

We carefully characterized the functions and pathways of different states of PTCs by gene enrichment analysiS (Fig. 2H, Supplemental Fig. S7C). The injured PTCs lose most of the physiological functions of the renal tubules, including the reabsorption of water and various ion substances, and instead activate a variety of different inflammatory pathways. We noted that Ferroptosis was activated in three clusters of injured PTCs. Given the interspersed presence of molecular expression patterns in multiple different cell death events [31–35], the activation of Ferroptosis implies that similar modes of death may be widely activated. We extracted key genes of Pyroptosis from the Gene ontology (GO) database and key genes of Necroptosis and Ferroptosis from the Kyoto Encyclopedia of Genes and Genomes (KEGG) database and scored the pathway activity by AUCell. The cell death pathway activity of the different cell types was assessed by scoring all cell types of the overall model. The results showed that Necroptosis had high pathway activity in most parenchymal and immune cells, more significantly in neutrophils, endothelial cells, T cells, dendritic cells, and macrophages (Fig. 3A, Supplemental Fig. S8A). Pyroptosis had high pathway activity in specific immune cells, especially in macrophages, NK cells, and neutrophils, which is consistent with the existing knowledge [36], while PTCs had the lowest pathway activity of all cell types (Fig. 3B, Supplemental Fig. S8B). Ferroptosis showed high pathway activity in different segments of renal tubular cells, macrophages and neutrophils (Fig. 3C, Supplemental Fig. S8C).

PTCs are one of the critical cells in AKI kidney injury, and numerous studies have focused on them in recent years. We isolated PTCs from the overall model to compare the pathway activity of Necroptosis, Pyroptosis and Ferroptosis among PTCs in different models. The results showed that the pathway activity of Necroptosis was significantly upregulated in PTCs of CP, UUO,IRI and SO models compared to the Control group (*P* < 0.05), while there was no significant difference in the FA model (Fig. 3D). For the Pyroptosis pathway, the activity of pathway was upregulated in PTCs of the UUO and IRI models compared with the Control group (*P* < 0.05), while the pathway activity was downregulated in the CP and SO models (*P* < 0.05) and not significantly different in the FA model (Fig. 3E), indicating that Pyroptosis may be activated only in PTCs of the UUO and IRI models, which we speculate may be caused by Maladaptive-PTCs sharing the killing spectrum with immune cells [37]. The results of Ferroptosis pathway activity suggested that Ferroptosis pathway activity was significantly upregulated in PTCs of different AKI models (*P* < 0.05) (Fig. 3F). Subsequently, by the key markers of Necroptosis, Ferroptosis and Pyroptosis, we verified the heterogeneity of cell death events in different model (Fig. 3G, H and Supplemental Fig. S9, S10). In the discussion section, we thoroughly discuss the relationship between different models of PTCs and different forms of regulated death modalities. Specifically intervening in one or more cell death pathways in different models might be effective in stopping AKI progression.

### Transcriptional trajectories from healthy to injured PTCs and proteomics analysis revealed extensive cytoskeletal remodeling events in AKI-PTCs

**Fig. 4:**
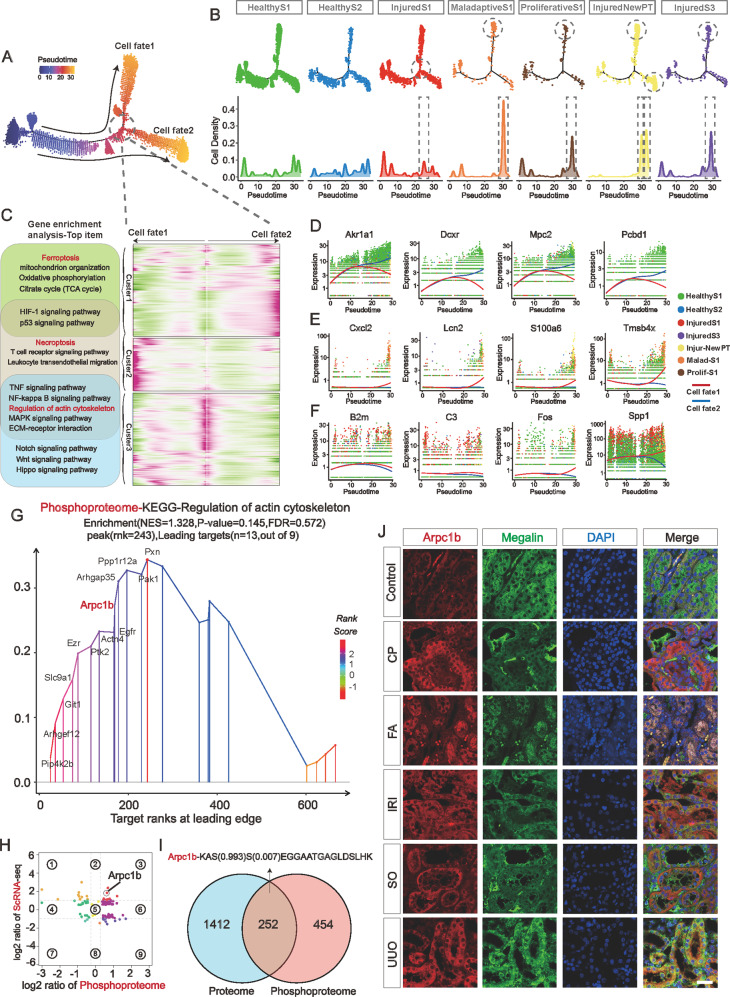
Transcriptional trajectories from healthy to injured PTCs and proteomics analysis revealed extensive cytoskeletal remodeling events in AKI-PTCs. **A** Pseudotime analysis of the differentiation trajectory of PTCs. PTCs gradually progressed to two cell fate as AKI progressed. **B** Cell number distribution of 7 PTC celltypes on the pseudotime trajectory. Maladaptive-PTCs, Proliferative PTCs and part of the *Krt20* NewPT were mainly distributed on the trajectory of cellfate1, and the remaining NewPT and InjuredS3 were mainly distributed on the trajectory of cellfate2. InjuredS1 PTCs were mainly distributed on the bifurcation of cellfate1 and cellfate2, indicating that these cells may be the earliest corrupted PTC cell subpopulation. **C** BEAM heatmap analysis identified genes associated with cellfate1 and cellfate2 differentiation. A total of three clusters are identified. The genes in Cluster1 are related to cellfate2 and Ferroptosis pathway were activated in this cluster. The genes in Cluster2 are related to cellfate1 and Necroptosis pathway were activated in this cluster. The Cluster3 lies at the bifurcation of the Pseudotime trajectory, and the heatmap reveals that a large number of genes are progressively highly expressed toward the Cellfate1 trajectory. Notably, the regulation of the cytoskeleton is a widespread feature of the PTC injury process. **D**–**F** Expression of Top genes of Cluster1 (**D**), Cluster2 (**E**), and Cluster3 (**F**) in the BEAM heatmap. **G** GSEA analysis of differentially expressed proteins in the phosphoproteomics, and the most significantly enriched pathway in the KEGG was the regulation of actin cytoskeleton pathway, in which Arpc1b is also involved. **H**, **I** In a combined analysis of the UUO mouse kidney proteomics and single-cell sequencing data of AKI-PTCs, a protein was identified that was simultaneously highly expressed in transcriptional data, quantitative protein data, and phosphorylated protein data: Arpc1b, and its phosphorylation site was determined. **J** Co-staining of sections from Control, cisplatin (CP), folic acid (FA), sodium oxalate (SO), ischemia reperfusion injury (IRI), and unilateral ureteral obstruction (UUO) kidneys for Arpc1b (red) and Megalin (green). Scale bars = 100 μM. Quantitative analysis of protein immunofluorescence intensity in Supplemental Fig. S2E.

To uncover the details of the differentiation of inflammatory state PTCs, we performed pseudotime extrapolation to the differentiation trajectory of PTCs. monocle analysis revealed that the injured and maladaptive PTCs were at the end of the differentiation trajectory and that maladaptive differentiated separately from one another (cellfate2). InjuredNewPT (*Krt20*) is scattered in the terminal branches of cellfate1 and cellfate2. Interestingly, InjuredS1 PTCs were at the intersection node of cellfate1 and cellfate2 differentiation branch (Fig. 4A, B). We performed a BEAM analysis of the nodes at the differentiation bifurcation **(**Fig. 4C, Supplemental Table S4). The three clusters of the BEAM heatmap represent cellfate1, 2 and intermediate nodes respectively. We analyzed the pathways and functions associated with the differentiation of PTCs (Fig. 4C), and combined with gene enrichment analysis of PTCs in different functional states (Fig. 2H, Supplemental Fig. S7C), we observed the prevalence of positive and negative regulatory events of cytoskeletal remodeling during the process of injury and differentiation of AKI-PTCs. We further visualized the Top expressed genes of the three clusters of the BEAM heatmap. The Top genes of Cluster contained several important genes related to kidney injury, such as *Akr1a1* [38], *Cxcl2*, *Lcn2*, *C3*, et al. Interestingly, we observed that the protein Tmsb4x, which is known to be involved in cytoskeletal remodeling [39, 40], was closely associated with the differentiation of Cellfate1 (Fig. 4D). By immunofluorescence and ELISA, we verified the expression of Tmsb4x in PTCs and mouse urine (Supplemental Fig. S11).

In addition, In the phosphorylated proteomic data of UUO-AKI, regulation of actin cytoskeleton was enriched as the top entry for GSEA analysis, again suggesting the prevalence of cytoskeletal remodeling events in AKI (Fig. 4G). Furthermore, by co-analysis of single-cell sequencing data of AKI-PTCs with UUO mouse proteome and phosphorylated proteomics data, we identified a cytoskeleton remodeling-associated protein Arpc1b that was significantly upregulated at both transcriptional, translational, and post-translational modification levels (Fig. 4H, I and Supplemental Fig. S12A–D). Immunofluorescence and ELISA also confirmed the expression of Arpc1b in AKI (Fig. 4J and Supplemental Fig. S12 E, F). In addition, both *Tmsb4x* and *Arpc1b* showed good diagnostic performance in two independent AKI datasets **(**Supplemental Fig. S13). These results indicate the prevalence of cytoskeletal remodeling events in AKI, especially in PTCs. Considering its specific biological function, intervention in this pathway might improve the poor prognosis caused by morphological dysregulation of AKI-PTCs. However, as only kidneys from UUO mice were used for proteomic and phosphorylated proteomic analyses, conclusions on cytoskeletal remodeling events may be limited and additional data from more models are required for further studies.

### Unsupervised screening strategies identified 18 biomarkers specifically expressed in AKI-PTCs

**Fig. 5:**
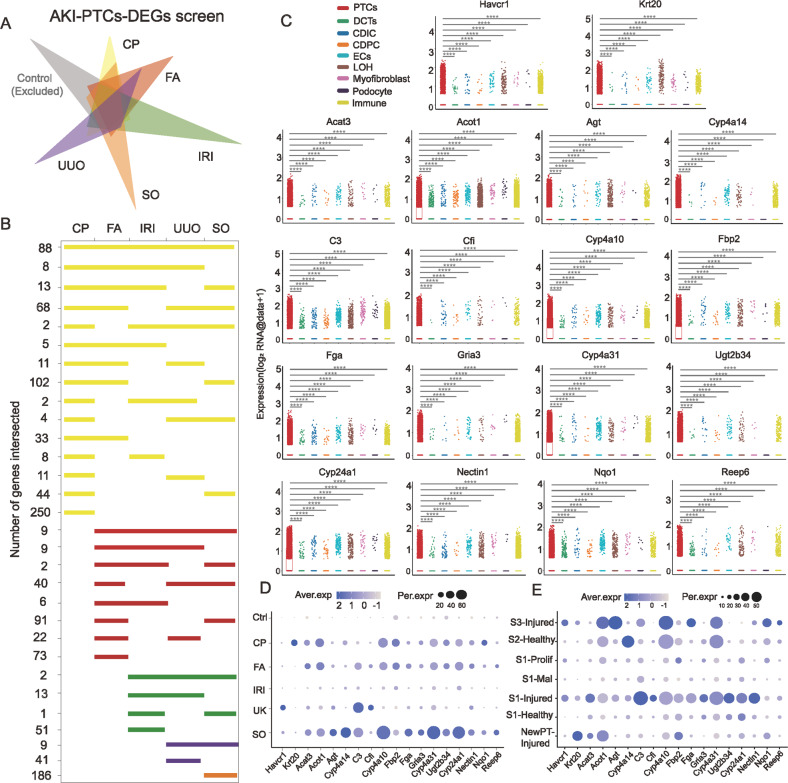
Identification of specific biomarkers for PTCs in single or multiple AKI models through unsupervised screening strategies. **A** Identification of differentially expressed genes (DEGs) specifically expressed in AKI-PTCs by the strategy of Supplemental Fig. S14A. The aim was to screen for DEGs specifically expressed in different etiologically induced AKI, which required limiting the expression of Control. **B** After excluding genes intersected with Control, the number of intersections of PTCs-DEGs for each model. **C** A total of 18 genes that were specifically highly expressed in PTCs of the AKI dataset were screened. Genes including *Havcr1*, *C3*, and *Krt20* were identified in the unsupervised setting, which confirmed the reliability of the screening strategy. Kruskal–Wallis test, ****P < 0.0001 vs. PTCs group. **D** Expression of 18 DEGs in different models of PTCs. **E** Expression of 18 DEGs in seven different states of PTCs.

Identification of injury-state PTCs in AKI at an early stage is significant to intervene the progression of AKI, currently the relevant biomarkers are still scarce and often species-limited. Through a series of unsupervised screening strategies (Fig. 5A, B**;** Supplemental Fig. S14A), we obtained 18 genes that are uniquely highly expressed in AKI-PTCs (Fig. 5C–E). Both *Havcr1* [41] and *Krt20* [30], known to be classical markers of PTCs injury, were screened by this unsupervised screening strategy. We validated the expression of these genes in two independent bulk datasets (IRI and CP) and two single-cell validation datasets (IRI and UUO) (Supplemental Fig. S14B, C, S15). Clinical data from the Nephroseq database indicate that most of these genes were correlated with poor prognosis of kidney disease (Supplemental Fig. S14D). Notably, some of the genes are of murine origin (*Acat3*, *Cyp4a31*, *Cyp4a10*, *Cyp4a14*, *Ugt2b34*, *Nectin1*), highly expressed only in mice, and have the potential to become biomarkers in mice kidney injury studies. We isolated PTCs from each AKI model and verified the expression of these six genes using Real-time PCR, and the results were consistent with scRNA data (Supplemental Fig. S16).

### Identification *S100a6* as a novel biomarker for inflamed endothelial cells in a multi-model AKI

**Fig. 6:**
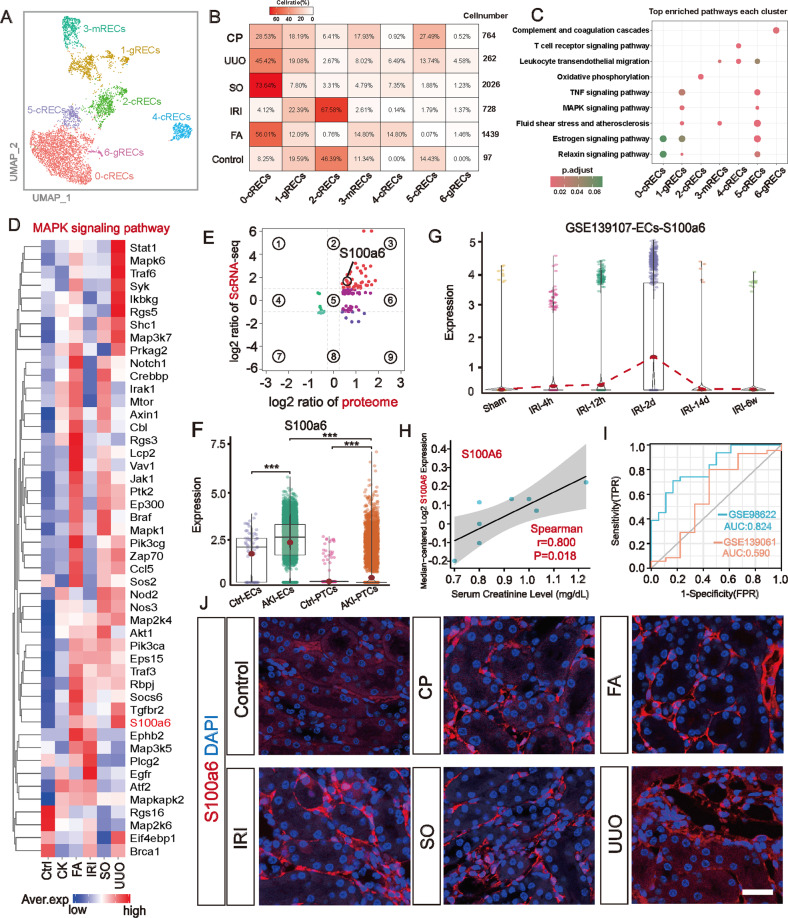
Identification of *S100a6* in the MAPK signaling pathway as a novel biomarker for inflamed AKI-ECs. **A** In combination with known renal EC biomarkers (Supplemental Fig. S17A) for cell type identification of separated ECs, a total of 6 subclusters with 3 distinct EC subpopulations were identified. **B** Compared to the Control model, the AKI model identified more ECs, and the UUO, FA and SO models were closer in subpopulation proportions compared to the IRI and CP models. **C** The top KEGG pathways enriched to each ECs subpopulation showed that immune and inflammation-related pathways were mainly occurring in Clsuter1, 3, 4, 5, and 6, involving all three endothelial cell subtypes, and the classical TNF and MAPK signaling pathways were activated. **D** The MAPK signaling pathway in AKI-ECs, with *S100a6* being one of the activating genes of this pathway. **E** The DEGs of ECs1-6 were correlated with the proteome of UUO, and *S100a6* was upregulated at both transcriptional and translational levels. **F** *S100a6* was more significantly expressed in ECs compared to PTCs. Kruskal-Wallis test, ***P < 0.001 vs. Ctrl group. **G** In the IRI multi-time-point snRNA-seq dataset (GSE139107), *S100a6* was upregulated at the second day after kidney injury and declined subsequently. **H** High expression of *S100a6* was correlated with high levels of creatinine. **I** Through ROC diagnostic curve, the diagnostic efficacy of *S100a6* in differentiating diseases was tested in the AKI public datasets GSE98622 (AUC:0.824, CI:0.707-0.942) and GSE139061 (AUC:0.590, CI:0.338-0.841). **J** Immunofluorescence staining validates S100a6 (red) in AKI-ECs, Scale bars = 100 μM. Quantitative analysis of protein immunofluorescence intensity in Supplemental Fig. S7C.

In scRNA-seq, ECs are more difficult to identified as they are more embedded in the extracellular matrix [42]. We separated ECs for re-clustering. The results showed that for ECs, the secondary clustering improved the accuracy of cell type identification. We identified three different types of endothelial cells [43]: cRECs (cortical renal endothelial cells, *Npr3Itgfbp3*), mRECs (medullary renal endothelial cells, *Aqp1Itgfbp7*), and gRECs (glomeruli renal endothelial cells, *Tspan7Pi16*) (Fig. 6A; Supplemental Fig. S17A, B and Supplemental Table S5). Notably, we observed a much higher number of ECs in AKI models compared to Controls, and the UUO, FA and SO models were much closer in terms of subcluster cell proportions (Fig. 6B). Since there were almost no batch effects for ECs in this dataset, the clustering of different subclusters of ECs relied on the biological differences between them. We inferred the specific status of each ECs subcluster by the top pathways enriched in that subcluster (Fig. 6C). The results showed that ECs of sublusters 0 and 2 were barely involved in immune and inflammatory pathways. In contrast, ECs of subclusters 1, 3, 4, 5, and 6 were involved in some classical inflammatory and immune signaling pathways, which included the TNF signaling pathway and MAPK signaling pathway. Heatmap analysis comfirmed key genes of this pathway were largely activated in different AKI-ECs (Fig. 6D). Furthermore, by combining proteomic data, we found that a gene in this pathway, *S100a6*, and its encoded protein were consistently highly expressed at the transcriptional and translational levels (Fig. 6E). Although a previous study by cheng et al. [44]. had developed *S100a6* as a marker of tubular injury and recovery during AKI, we found that *S100a6* was more significantly expressed in ECs compared to PTCs (Fig. 6F), and its distinctive upregulation during AKI endows its potentiality as a unique AKI-EC marker (Fig. 6G). Correlation analysis with clinical data demonstrated that high expression of *S100a6* was associated with high creatinine levels (Fig. 6H), and in addition, *S100a6* showed good diagnostic efficacy as a marker of impairment in two AKI public datasets (Fig. 6I). Subsequently, we verified S100a6 protein expression in different AKI models by immunofluorescence and ELISA (Fig. 6J and Supplemental Fig. S17 C, D).

### WGCNA revealed DCTs, LOH, CDIC, and CDPC were engaged in milder inflammatory processes like apoptosis and autophagy

**Fig. 7:**
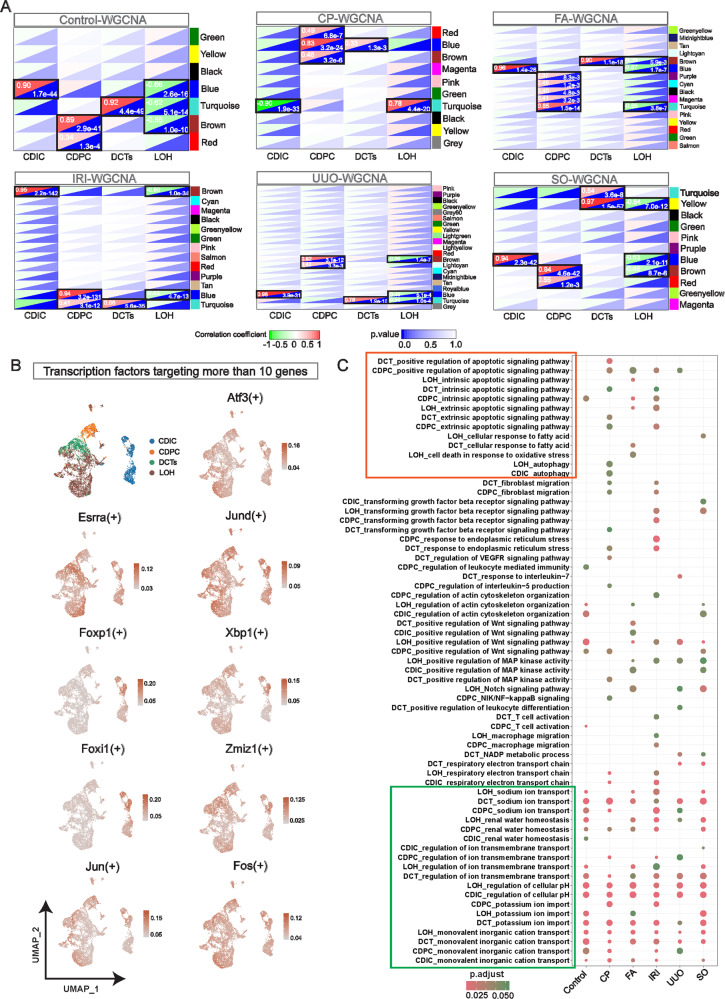
WGCNA revealed the mild trans-model injury characteristics of DCT, LOH, CDIC and CDPC. **A** In Control and 5 AKI models, randomly selected cells from DCTs, LOH, CDIC, and CDPC were selected for Weighted gene correlation network analysis (WGCNA) analysis using the method of Pseudocell. Key modules (black boxes) and key genes associated with cell types were screened based on correlation coefficients and *p* values. **B** The expression of 9 transcription factors in 4 cell types. Each of these 9 transcription factors regulates more than 10 key genes involved in basic cellular functional homeostasis (ion transport, water homeostasis, PH regulation) of DCTs, LOH, CDIC, and CDPC (panel C). Among them, *Atf3*, *Jun*, *Fos*, *Jund* and *Zmiz1* are also involved in the regulation of most key genes related to apoptosis and autophagy. **C** Enrichment analysis of integrated biological processes revealed that DCT, LOH, CDIC, and CDPC in early AKI are not involved in drastic forms of inflammatory processes. In contrast, apoptosis, autophagy, and other pathways are activated. Cells in these four segments retained regular tubular functions in different models (green boxes), including reabsorption of various metal ions and regulation of water homeostasis.

The injury pattern of renal tubular segments following PTCs in AKI is poorly understood. We identified the key modules and the key genes of DCTs, LOH, CDIC, and CDPC in Control and each AKI model by Weighted gene co-expression network (WGCNA) (Fig. 7A**;** Supplemental Fig. S18A and Supplemental Table S6). Through gene ontology, we identify the biological processes in which these four cell types are mainly involved in each model. Interestingly, after the onset of AKI, these four segments of renal tubules are not involved in drastic forms of inflammation and injury-related biological processes. The fundamental renal tubular functions including water homeostasis, metal ion transport and, PH regulation are largely preserved in all AKI models (Fig. 7B). In contrast to PTCs, relatively mild autophagy and apoptosis pathways are widely activated. To identify transcription factors that regulate these essential functions and the autophagic, apoptotic pathway, we performed a SCENCI analysis. Nine key TFs (*Atf3*, *Esrra*, *Jund*, *Foxp1*, *Xbp1*, *Foxi1*, *Zmiz1*, Jun and *Fos*) regulated the essential fundamental functions, five of which (*Atf3*, *Jund*, *Xbp1*, *Zmiz1*, and *Fos*) simultaneously regulate most genes of the apoptosis and autophagy pathways (Fig. 7C, and Supplemental Fig. S18B).

### Dynamically active immune-parenchymal intercellular communication patterns are important features of AKI with different etiologies

**Fig. 8:**
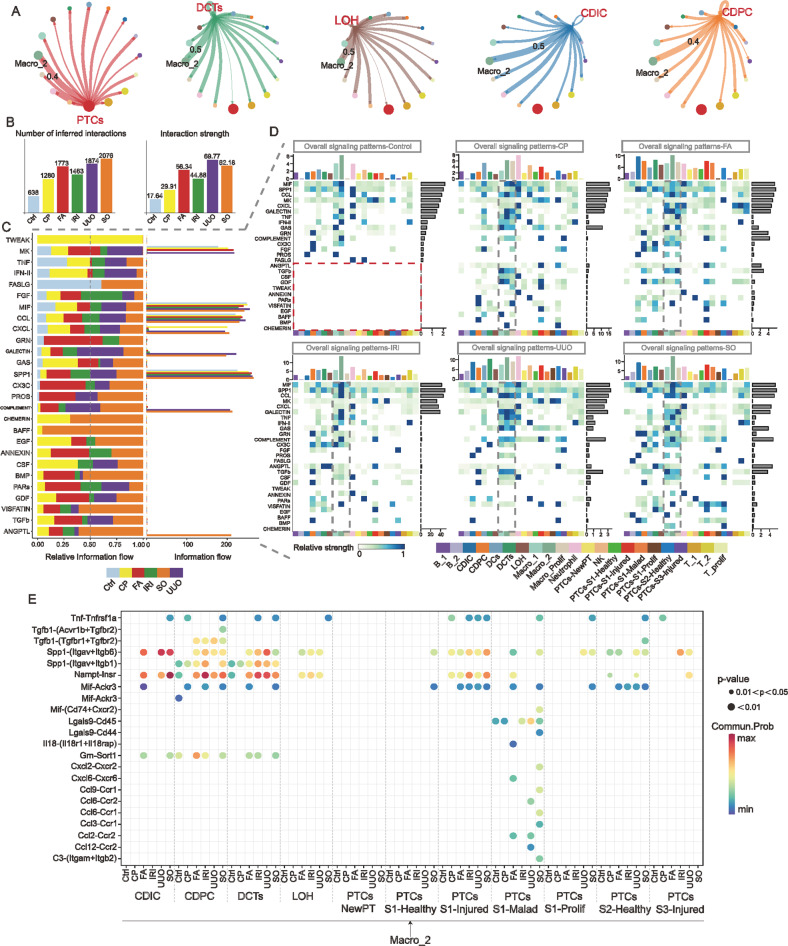
*Arg1* Macro_2 cells have dynamically active intercellular ligand-receptor interactions with AKI parenchyma cells. **A** In the immune-parenchymal cell communication network of the integrated AKI dataset, *Arg1* Macro_2 cells (Fig. 1B) were the immune cell subtype with the highest interaction weight with consecutive renal tubular segments. **B** The number and weight of the connections for intercellular communication are increased in AKI with different etiology compared to Control. The active pattern of intercellular interaction within AKI is one of the characteristics of AKI. **C** Differences in the overall information flow of the 27 selected secretory intercellular ligand-receptor signaling pathways in different AKI models. The information flow was calculated from the sum of the communication probabilities between all pairs of cell populations in the inferred network (i.e., the total weight in the network). **D** Overall signaling heatmap of 27 selected outgoing and incoming signals for all immune cell subtypes, PTCs subtypes, DCTs, LOH, CDIC, and CDPC in Control and each AKI model. Compared to Control, partial intercellular signaling interaction events were only enriched in several AKI models (The red dashed box in Control). Signaling events of macrophage subtypes were specifically prominent in immune cells across different models (Gray dashed box). **E** Ligand-receptors with *p* values <0.05 in *Arg1* Macro_2 cells with each segment of renal tubular parenchyma cells in Control and each AKI model.

In pathological conditions, the cellular status is simultaneously impacted by its own internal and peripheral immune cells. Compared to Control, intercellular communication is noticeably active after AKI onset (Fig. 8B, and Supplemental Fig. S19A). Among all immune cell subtypes, *Arg1* Macro_2 cells (Fig. 1B) are the immune cells that interact most closely with different renal tubule segments, which possesses the highest interaction weight in all AKI models (Fig. 8A). Next, we thoroughly analyzed the information flow patterns of 27 secretory intercellular interaction signaling pathways in different models. In contrast to Control, some intercellular communication signals are specifically expressed in AKI of different etiologies, for example, TWEAK signaling is highly active in cisplatin-induced AKI and BAFF signaling pathway is highly active in SO-induced AKI (Fig. 8C). Notably, the overall signal heatmap similarly suggests that macrophages and its subtypes are the most active immune cell subtypes (Fig. 8D). In particular, specific ligand-receptor signals in TNF, SPP1, MIF, VISFATIN, and TGFb signaling pathways were significantly enriched between *Arg1* Macro_2 cells and renal tubule cells in multiple models (Fig. 8E). Additional schematic heatmaps reveal that macrophages act as major senders, receivers, regulators and influencers of ligand-receptor signals that affect parenchymal cells (Supplemental Fig. S19B, C). Based on these specific ligand-receptor signaling patterns, intervening in the ligand-receptor linkage between Macro_2 cells and parenchymal cells in different AKI models might ameliorate immune inflammation-induced kidney damage.

## Discussion

We hypothesize that an in-depth comparison of mechanistic causes of AKI progression induced by different etiologies will aid in the development of more accurate diagnostics, treatments, and preventative strategies against AKI. scRNA-seq provides an unprecedented high-resolution view of the similarities and differences in different etiologies-induced AKI. By scRNA-seq, we found that FA- and SO-induced AKI and UUO-induced obstructive AKI were closer in immune and injured parenchymal cell ratios (Figs. 1D, 2G, and 6B). Previous findings indicated that FA-, UUO-, and SO-induced kidney injury exhibits more typical features of chronicized renal injury as time progresses, including pathological changes of collagen deposition and interstitial renal fibrosis [9]. Our results showed that homogenized injury features among different models may have emerged at the early stages of kidney injury. From a pathologic point of view, both the FA and the SO lead to the formation of crystals in the PTCs resulting in renal tubule blockage and subsequent pathological changes similar to those of obstructive nephropathy. In contrast, the CP and IRI models exhibited unique injury characteristics including different injury outcomes in PTCs. These data suggested careful consideration of the impact factors of different AKI would influence the development of novel therapies for AKI.

Our analysis highlights the characteristics of different renal tubular segments during AKI. The results indicated that proximal tubules do represent the most vulnerable tubular cells compared to DCTs, LOH, CDIC, and CDPC [45]. And our studies on PTCs subtypes have strengthened the understanding of *Krt20* PTCs cells and subpopulations of pro-inflammatory and pro-fibrotic maladaptive-PTCs in different AKI models. Krt20, a member of the keratin family, is an intermediate filament protein responsible for the structural integrity of epithelial cells, and we hypothesized that the presence of high expression of Krt20 cells in the kidney represents the disruption of epithelial cell integrity, which may be a transitional state in the injury process of PTCs [30].

We explored the pathway activity of Necroptosis, Pyroptosis and Ferroptosis in different cell types by single-cell sequencing. We noticed that immune cells, especially macrophages and neutrophils, had stronger cell death pathway activity compared to parenchymal cells, which reminds us of the need to focus on the role of programmed necrosis of immune cells in the renal injury process despite the fact that PTCs are one of the core cells in the renal injury process [37]. In addition, our results of isolating PTCs for re-scoring were broadly consistent with previous studies [46]. For example, Linkermann et al. validated both Necroptosis and Ferroptosis exist in IRI-AKI [47, 48], our previous work validated necroptosis in CP-AKI [21], Mulay et al. validated necroptosis in SO-AKI [49]. In UUO-AKI, some comprehensive studies demonstrated that all three forms of cell death co-exist [28, 50, 51]. In FA-AKI, more in-depth studies have also demonstrated that it is Ferroptosis rather than Necroptosis was the predominant form of cell death [22]. Furthermore, Balzer et al. expanded *Gsdmd* as a biomarker of injury in IRI-PTCs [15], and our previous study showed that upregulation of *Gsdme* in UUO-PTCs would results in the development of Pyroptosis [25]. However, compared to other cell types, Pyroptosis pathway activity in PTCs is at an extremely low level, and the expression of GSDMD and GSDME does not appear to be significant in most PTCs in AKI models. A study has confirmed the non-cell autonomous role of GSDMD in protecting renal tubules from necroptosis-mediated injury in IRI [52], and these results suggest that further studies on scorch death need to focus on the crosstalk between different modes of death and pyroptosis occurring in immune cells. Another non-negligible observation is that two or even multiple cell death pathways may be activated simultaneously in PTCs. Jointly blocking Necroptosis and Pyroptosis, and even Ferroptosis may provide a better strategy to halt the progression of AKI, as partially confirmed in our previous study [53, 54]. Interestingly, we discovered that cytoskeletal remodeling co-exists with PTCs injury, and high expression of genes and proteins related to skeletal remodeling is associated with a poor prognosis of AKI, which may become important biological markers to predict the prognosis of renal injury.

There are enriched and stable ligand-receptor communications between different immune cell subpopulations and parenchymal cells in AKI kidneys, and this pattern of cellular communication is not greatly biased by differences in cell ratios between models. Macrophage, especially *Arg1* Macro_2 cells (Figs. 1B and 7A) are always the very important immune cells that interact with other cells. According to the expression profile and cell proportions, Macro_2 cells correspond to M2 macrophages. Previous studies have demonstrated that M1 macrophages trigger kidney injury by releasing pro-inflammatory cytokines such as IL-6, TNFα and IL-1β, while M2 macrophages play an anti-inflammatory role in the inflammatory process [55, 56]. Different types of macrophages coexist in different disease stages of renal inflammation, repair and fibrosis, but the M1/M2 ratio changes over time. In scRNA-seq, macrophages drive the renal injury process by interacting with parenchymal cells through multiple well-known pro-inflammatory and pro-fibrotic pathways as bridges. Although Macro_2 was the most prominent immune cell in our data, it should not be ignored that Macro_1 cells had equally robust signaling intensity in CP, FA and UUO models, with active intercellular TGFb, MIF and CCL signaling being common features of both Macro_2 and Macro_1 cells [57]. Our results suggest that the switching of different subtypes of macrophages is initiated at the early stage of kidney injury, and although the specific details of the transition are not yet clear, a deeper exploration of the signaling bridges may be a novel entry point to study the switching of M1/M2 macrophage states and the pathogenic role of M2 cells during kidney injury. Since AKI corresponds to the early stages of the disease, where the overall immune cell status is in a delicate balance of pro- and anti-inflammatory. Activated M2 macrophages would imply that renal tissues are exerting an active compensatory role for injury. However, in the absence of blockade of the etiology or further intervention, the anti-inflammatory and pro-repair M2 macrophages often lead to abnormal tissue repair and promote the development of chronic fibrosis [37, 58]. Inflammation and fibrosis are known compensatory repair mechanisms, AKI will progress to chronicity when the balance between them is disturbed. An early intervention targeting *Arg1* macrophages may be one of the key components in arresting the chronicity of AKI.

Overall, our study provides a very comprehensive single-cell atlas of multi-model AKI to date, which will provide a valuable and important resource for future studies of the intrinsic mechanisms and therapeutic targets of AKI and facilitate future insights into the underlying regulatory mechanisms of each cell type.

## Methods

### Mice

C57BL/6 mice (8–10 weeks, 23–26 g, male) were obtained from the pathogen-free (SPF) facility of Fujian Medical University. All animal experiments were approved by the Laboratory Animal Management and Ethics Committee of Fujian Medical University and were performed in accordance with the “China Guide for the Protection and Use of Laboratory Animals”. All mice were housed in a specific pathogen-free facility with a 12-hour light/dark cycle.

### Induction of acute kidney injury model

Male mice were used in all these models, and four kidneys from two mice were used for single-cell sequencing in each model. For IRI and UUO-induced AKI, the procedures were performed by a skillful surgeon in a temperature-controlled room (25 °C). For IRI model, retroperitoneal clipping surgery was used. After mice were anesthetized with ketamine (80–100 mg/kg/i.p., Cayman Chemical) and xylazine (10 mg/kg/i.p., Selleck Chemicals), approximate 10-mm incisions were performed at a distance of about 8 mm on each side of the spine. After both of kidneys were carefully exposed, bilateral renal pedicles were clamped for 43 min by a vascular clip (Fine Science Tools). The kidney was observed visually with a loss of blood supply and turning pale. During this period, the surgical region on the back of the mice was covered with saline gauze and the kidney was kept in wet condition with normal saline. After the vascular clamps were removed to restore the blood supply with visually reperfusion, the surgical incisions were closed in two layers with 5-0 sutures. The mice were then injected with pre-warmed physiological saline solution (37 °C; 1 ml per 20 g body weight) subcutaneously (s.c.). Throughout the surgical procedure, the body temperature was maintained between 36 °C and 37 °C by continuous monitoring using a Homeothermic System (Harvard Apparatus) until they recovered from anesthesia. At the end of these procedures, mice were put back in cages in a temperature-controlled room (25 °C) where free access to water and food was available. Mice were monitored closely. Pre- and postoperative analgesia (Ibuprofen, 200 μg/ml in drinking water) were given to the mice from 24 h before and until 48 h after surgery.

For CP-induced AKI, C57BL/6 male mice were given a single intraperitoneal injection of cisplatin (Hansoh Pharma, China) at a dose of 20 mg/kg. For acute crystal nephropathies, we used FA or SO-induced AKI. For FA-induced AKI, male mice received a single intraperitoneal injection of 250 mg/kg folic acid (Sigma-Aldrich) in 0.3 mol/L sodium bicarbonate. For oxalate-induced AKI, male mice were given with a single intraperitoneal injection of 100 mg/kg sodium oxalate (Sigma) and 3% sodium oxalate in drinking water.

For UUO-induced acute obstructive AKI, as previously described [25], C57BL/6 male mice were anesthetized as mentioned above. The unilateral ureter ligation was also performed from retroperitoneal pathway. Briefly, an about 10-mm of incision was made on the left side of the spine, the kidney was carefully exposed and then followed by blunt separation of the ureter and peritoneum. Ureteral obstruction was performed by the ligation of the left ureter with 4-0 silk suture. Fluid resuscitation and care procedures for the mice are the same with IRI model.

All samples were harvested from the mice after 48 hours of treatment in CP, FA, SO and IRI-induced AKI model. The obstructive kidney in the UUO model were collected after 2 days. The pathological injury score of the injury kidneys in these models without no statistical difference were selected for single-cell sequencing analysis.

### Preparation of kidney single-cell suspensions

Kidneys were placed on ice-cold Dulbecco’s phosphate buffered saline (DPBS), debrided, cut longitudinally, and coronally sectioned. The renal pelvis portion of the kidney was then excised with a sterile razor, and the remaining tissue was washed in a 6 cm Petri dish with 1× DPBS to remove residues such as blood stains. Subsequently, the tissue was sheared to a size of 5 × 5 mm^3^. The cleaned samples were then transferred to 5 mL centrifuge tubes with 1× DPBS and placed on ice to rapidly shear the tissues to 1–3 mm^3^. Next, 10 times the volume of 1× DPBS was added to the sheared tissues which were cleaned by gentle blowing with a Pap sieve five times, and collected by filtering through a 70 μm cell sieve. The collected tissues were transferred into the prepared digestive enzyme solution (collagen II 2 mg/mL, collagen IV 2 mg/mL) and incubated for 30 min at 37 °C, 100 rpm; the dissociated solution mixed with tissues and cells was transferred into 2–3 times the volume of 1× DPBS, washed by blowing with a Bass pipette and passed through a 40 μm cell sieve before the filtrate was collected. The filtered cell suspension was centrifuged at 300 × *g* for 5 min at 12 °C. The supernatant was discarded and the remaining cells were collected. Any erythrocytes remaining in the cell fractions were removed by lysis in erythrocyte lysate for 3–10 min. Subsequently, the precipitate was resuspended in 1× DPBS using 10 times the volume of cell suspension used in the previous step, washed 1–2 times, centrifuged again as previously described (300 × *g* at 12 °C for 5 min). Cell densities were adjusted to 700–1200 cells/μL with 1640 (+5% (v/v) fetal bovine serum (FBS) before analysis.

### Single-cell library generation and sequencing

Harvested cells at the required densities were combined with gel beads containing the barcoded information along with a mixture of cells and enzymes. Oil surfactant droplets in a microfluidic “double-cross” system were used for encapsulation to generate Gel Beads-In-Emulsions (GEMs). GEMs were passed through a reservoir and collected while the gel beads were lysed to release the barcode sequence. The cDNA fragment was then reverse transcribed before the sample was labeled. The gel beads and the oil droplets are ruptured and PCR amplification was performed using the generated cDNA as a template. The products of all GEMs were mixed and a standard sequencing library is constructed. The cDNA was first enzymatically digested into fragments of about 200–300 base pairs (bp), together with the library building process of traditional second-generation sequencing such as sequencing junction and primers. Finally, the DNA library was generated by PCR amplification. Cell capture was performed using the official library kit (10X Genomics Chromium Single-Cell 3’ kit, V3) according to manufacturer’s instructions. Following the capture of 10,000 target cells, sequencing was performed using the NovaSeq6000 sequencing platform (paired-end multiplexing run, 150 bp) by LC-Bio Technology Co. Ltd. (Hangzhou, China). A sequencing depth of 50,000 reads per cell was required.

### Quality control of single-cell sequencing data

Sequencing data were parsed using Illumina bcl2fastq software (Version 2.20) and converted to FASTQ format files. The cleaned FASTQ files were processed using cellranger (Version 6.0). SoupX [59] (Version 1.6.1) package was used to correct ambient RNA contamination and the Seurat [60, 61] (Version 4.2.0) package was used for downstream analyses. The following parameters were used to remove low-quality cells: (1) exclude cells expressing ≤500 or ≥4000 genes/cell; (2) exclude cells expressing ≤500 or ≥15000 unique molecular identifier per cell (UMIs/cell); (3) exclude cells with high cell complexity (log10GenesPerUMI) ≤0.8; (4) due to the fact that renal tubular epithelial cells in the kidney are very energetically involved in active transport, which requires a large number of mitochondria to provide energy [62, 63]. We exclude cells with >30% mitochondrial ratio; (5) exclude doublets using the DoubletFinder [64] (Version 2.0.3) package; (6) retain genes expressed in at least 10 cells. Since we were not interested in genes found in the ribosome or in erythrocytes, these genes were excluded from subsequent analyses. The LogNormalize method of the “Normalization” function was used for expression homogenization. The “FindVariableGenesfunction” function selected 2000 highly variable genes based on the average expression and dispersion of each gene. Since cell cycle arrest is a normal manifestation of AKI pathology, we did not regress the effect of cell cycle genes on the results.

### Unsupervised dimensionality reduction, removal of batch effects, and cell type identification

Following quality control analysis, we obtained high-quality single-cell data, which were subsequently analyzed by principal component analysis (PCA) using the RunPCA function for highly variable genes. The number of principal components (PCs) was selected based on three criteria: (1) the cumulative contribution of the PCs was >90%; (2) the PCs themselves contributed <5% to each other’s variance; and (3) the difference between two consecutive PCs was less than 0.1%. Biases caused by different batches were then removed using the harmony [65] package (version 0.1.0) and cells were clustered using the FindClusters function with resolution adjusted to 0.5–1.5, to exclude abnormal clusters that were independently present in duplicate samples of the AKI model and had <50 cells. Wilcoxon rank sum tests were performed using the FindMarkers function and min.pct was set to 0.25 (i.e., genes expressed in at least 25% of cells within or outside the cluster). Marker genes for each cluster were screened by differential expression analysis between cells inside and outside the cluster. Cell type annotation of the cell clusters was performed following comparison with classical cell type markers. The Subset function was used to isolate the specified cell types, and the above steps were subsequently repeated to remove batch effects and identify clusters driven by biological differences.

### Cell trajectory analysis

Cell developmental trajectory inference was performed using monocle [66] (Version 2.18.0) package. Raw read counts were used as input, specifying the expressionFamily parameter of the newCellDataSet function as a negative binomial distribution. The size factor and dispersions were estimated using the estimateSizeFactors and estimateDispersions. Construct cell trajectories were selected by monocle using highly variable genes. We considered the state with the most control cells as the root state and used the orderCells function to sort cells according to their actual state. The BEAM function was used to calculate key genes associated with the direction of cellular outcomes, and cells expressed in at least 100 cells were screened for analysis. The plot_genes_branched_heatmap function was used to select the top 2000 genes associated with development for data visualization. In contrast, the plot_genes_branched_pseudotime function was used to graphically visualize changes in individual genes over time. In the PTCs trajectory derivation, the above steps were repeated individually using the original PTCs counts for each model.

### Analysis of transcription factor regulatory networks

The SCENIC [67] (Version 1.2.4) and GENIE3 [68] (1.12.0) packages were used to infer potential transcriptional regulatory networks in DCTs, LOH, CDIC, and CDPC. The log-normalized expression matrix generated by Seurat was used as input data and all cells were randomly selected for analysis. After running GENIE3, the motif dataset (mm10_refseq-r80_500bp_up_and_100bp_down_tss.mc9nr.feather, mm10_refseq-r80_10kb_up_and_down_tss.mc9nr. feather) was used to construct each transcription factor. Co-expression modules between transcription factors and candidate target genes were inferred using GENIE3 (random forest), with each module containing a transcription factor and its target gene. Genes in each co-expression module were analyzed using RcisTarget to identify enriched motifs. Only TF motif-enriched modules and targets were retained and each TF and its potential direct target gene was referred to as a regulon. The activity of each regulon in each cell was assessed using AUCell [69] (version 1.16.0) to generate a score which was used to generate a regulon activity matrix that can be binarized (0|1, on|off) by setting an area under the curve (AUC) threshold for each regulon, which determines in which cells the regulon is in the “on” state.

### Analysis of intercellular ligand-receptor communication

Cell-to-cell communication analysis was performed using the CellChat [70] (version 1.5.0) package for all immune cell subtypes, PTCs subtypes, DCTs, LOH, CDIC and CDPC. A weighted directed network consisting of significant ligands between interacting cell groups is constructed by calculating the number of ligands and weight values of intercellular interactions. Subsequently, cellchat provided 2021 validated ligand-receptor signals for intermolecular interactions. Of these, the autocrine/paracrine related signal groups (which accounted for 61.8% of all ligand receptors) were selected to identify conserved and condition-specific signal information flow in Control and each AKI model. The signal strength was subsequently calculated in each cell type for the identified conserved and condition-specific signals. Outward and inward degrees were calculated in a weighted network as the sum of outgoing and incoming communication probabilities from the cell groups to identify the primary senders, receivers, mediators, and influencers of intercellular communication. And finally identification of ligand-receptors in specific high probability signals of specific cells.

### Cell-specific gene identification, gene enrichment analysis, specific pathway analysis, and clinical feature association analysis

Cell-specific genes in the Seurat run were calculated by FindAllMarkers according to metadata grouping. Genes with adj. *p* values less than 0.05 were screened, and each gene was ranked by the degree of difference using log2foldchange. Genes associated with the proposed temporal differentiation during the monocle run were calculated by the pData function. Gene enrichment analysis is a combination of GO and KEGG, using the clusterProfiler package and the Omicshare platform (https://www.omicshare.com/) for Gene Set Enrichment Analysis (GSEA) analysis. Extraction of key genes of specific pathways from GeneCards database (https://www.genecards.org/), counts values in single-cell sequencing of extracted genes for heatmap analysis. The kidney-related clinical characteristics data from the Nephroseq database (https://www.nephroseq.org/) were used for association analysis with specific differentially expressed genes (DEGs).

### Scoring of pathway activity for cell subpopulations

The Seurat’s AddModuleScore scoring method was used for pro-inflammatory and pro-fibrosis scoring of PTCs. The genes for the pro-inflammatory score incorporated 212 interleukin, TNF superfamily and chemokine genes. Genes for the pro-fibrosis score incorporated 73 TGFb pathway genes. The activity of three cell death pathways (Necroptosis, Pyroptosis, Ferroptosis) was scored by AUCell. Pathway genes for Necroptosis and Ferroptosis were collected from the GSEA database in the KEGG gene set. Pyroptosis-related genes were collected from the GO gene set of GSEA. Pathway genes were screened in combination with the reported relevant literature. The Score values of each cell were normalized or log(Score+1) processed and visualized using the ggplot2 package for analysis, and comparisons between groups were made using the Wilcoxon test.

### Public AKI dataset analysis

AKI-related datasets were identified in the Gene Expression Omnibus (GEO) database (https://www.ncbi.nlm.nih.gov/geo/) using “acute kidney injury” as the search keyword. GSE98622 [30] contained 18 kidney samples from sham mice and 31 from IRI-AKI mice while and GSE139061 [71] contained 9 kidney biopsy samples from healthy people and 39 from AKI patients. GSE165100 [72] contains 4 Control samples and 4 acute cisplatin-induced kidney injury sequencing data. The raw expression data of both datasets and their GPL platform files were downloaded, and the expression matrix was annotated using genesymbol. The expression of specific genes in the expression matrix was extracted, grouped according to the original research, and receiver operating characteristic (ROC) analysis was performed using the pROC (Version 1.18.0) package to calculate the AUC, with larger AUC values indicating greater sensitivity and specificity of the gene to as a predictor of disease. GSE139107 [13] contains renal single-cell sequencing data from 6 IRI time points and GSE140023 [73] contains renal single-cell sequencing data from UUO2 days and 7 days, both of these data were used to validate the expression of genes specific in PTCs.

### Weighted gene correlation network analysis (WGCNA)

The integrated scRNA dataset of all samples was sorted into DCTs, LOH, CDIC, and CDPC cell subpopulations by subset function. The normalized data values were extracted as the original matrix of WGCNA. The metadata file was extracted as the phenotype file of WGCNA. Using the Pseudocell method, set pseudocell=10, and randomly select a portion of cells from each cluster as the input matrix of WGCNA. Standardized WGCNA process analysis was performed via the sangerbox online platform. The standardized process included the construction of gene co-expression network, identification of gene modules, phenotype and modules for association analysis, and identification of key modules and key genes. The module merging threshold in the dynamic shear tree was 0.25, and the parameters for screening key genes were gene significance (GS) > 0.1 and module membership value (MM) > 0.7.

### Mass spectrometry-based proteomics and phosphorylated proteomics analyses

Wild-type (*n* = 3) and UUO-induced AKI mouse kidneys (*n* = 3) were stored at −80 °C and protein concentrations were determined using the bicinchoninic acid (BCA, Beyotime,China) kit. Equal amounts of each sample protein were taken for enzymatic digestion, and trypsin was added at a ratio of 1:50 (protease: protein, m/m) and incubated overnight. Dithiothreitol (DTT) was added to a final concentration of 5 mM and reduced at 56 °C for 30 min. Iodoacetamide (IAA) was added to a final concentration of 11 mM and incubated for 15 min at room temperature and protected from light. Peptides were dissolved in enrichment buffer solution (50% (v/v) acetonitrile/6% (v/v) trifluoroacetic acid). The supernatant was transferred to pre-washed immobilized metal affinity chromatography (IMAC) resin and incubated on a rotary shaker with gentle shaking. After incubation, the resin was washed three times with 50% (v/v) acetonitrile/6% (v/v) trifluoroacetic acid and 30% (v/v) acetonitrile/0.1% (v/v) trifluoroacetic acid buffer solutions. Finally, the modified peptides were eluted with 10% (v/v) ammonia before the eluate was collected, vacuum frozen, and dried. Peptides were desalted using C18 ZipTips according to the manufacturer’s instructions, before being vacuum freeze dried for liquid chromatography–mass spectrometry (LC-MS) analysis. Next, peptides were solubilized with liquid chromatography mobile phase A (0.1% (v/v) formic acid and 2% (v/v) acetonitrile) and separated using a NanoElute ultra high-performance liquid chromatography (UHPLC) system. Mobile phase B contained 0.1% (v/v) formic acid in 100% (v/v) acetonitrile. We used the following gradient method: 0–78 min, 2%–22% B; 78–84 min, 22%–35% B; 84–87 min, 35%–80% B; 87–90 min, 80% B. Peptides were separated by UHPLC and then injected into the capillary ion source for ionization and analyzed by timsTOF Pro mass spectrometry. The ion source voltage was set at 1.75 kV. Peptide parent ions and their secondary fragments were detected and analyzed using a high-resolution time of flight (TOF). The secondary mass spectrometry scan range was set to 400 – 1500 m/z. The parallel accumulated serial fragmentation (PASEF) mode was used for data acquisition. A primary mass spectrum acquisition was followed by 10 PASEF mode acquisitions of secondary spectra with parent ion charge numbers in the range of 0–5.

### Kidney histologic analysis in mice

Kidneys were embedded in paraffin or optimal cutting temperature compound (OCT, 4538, Leica). Paraffin sections (4 μm) were stained with periodic acid–Schiff (PAS) for the assessment of renal tubular injury. As previous studies [48], morphological damage of sections was evaluated by following parameters: brush border loss, tubular dilation, tubular necrosis and cast formation. The percentage of these parameters were counted on a scale of 0–10: 0, not present (normal); 1–4, 10~40% (mild); 5-6, 50~60% (moderate); 7–8, 70~80% (severe); 9–10, 90~100% (very severe).

Cryosections (4 μm) were used for immunofluorescence staining. Sections were washed with PBS after fixation with ice-cold acetone for 15 min, then incubated with the following different primary antibodies: Anti-TMSB4X (ab14334, 1:250; abcam), anti-ARPC1B (ab115217, 1:200; abcam), anti-S100A6 (ab181975, 1:200; abcam), anti-GSDMD (207701-1-AP, 1:250; Proteintech, China), anti-GSDME (13075-1-AP, 1:200, Proteintech, China), anti-RIP3 (sc-374639, 1:200; Santa Cruz), anti-4HNE (ab48506, 1:200; abcam), anti-NINJ1 (GTX31596, 1:50; GeneTex), anti-ZBP1 (sc-271483, 1:100; Santa Cruz), anti- KRT20 (ab97511, 1:200; abcam) and anti-Megalin (ab184676, 1:200; abcam) or anti-SLC34A1(ab151129, 1:200; abcam) antibody for 2–4 h. Both anti-Megalin antibody and anti-SLC34A1 antibody are PTC markers. As a detection antibody, AlexaFluor® 488 and Alexa Fluor® 594 labeled secondary antibodies (abcam) were used. Nucleus was labeled with DAPI (Invitrogen). All histologic sections were analyzed in a blinded manner. For morphologic quantifications, 3 random visual fields were analyzed per kidney section. The number of protein-positive cells were determined with Image J software. The normalized data were used as input files for Graphpad prism (Version 9.0) and statistically analyzed using One-way ANOVA.

### Enzyme linked immunosorbent assay (ELISA) for mouse urine

Mouse TMSB4X (EIAM-TMSB4X-1, RayBiotech), ARPC1B (ABX502254, BIOZOL), and S100A6 (ELM-S100A6-1, RayBiotech) ELISA Kit were used for detection of their urine levels by ELISA, respectively, in accordance with manufacturers’ instructions. And their concentrations were normalized per unit urinary volume.

### Flow cytometry analysis

Flow cytometry was used to quantify the immune cell subtypes in different AKI injured kidney. In brief, kidneys were harvested, minced, and incubated with 1 mg/ml type IV collagenase (sigma, 11088858001) and DnaseI (90083, ThermoFisher Scientific,) in DMEM (11965092, Gibco™) for 30 minutes at 37 °C in a shaker. The RPMI (31800-089, Gibco™) was added with FBS (085-150, WISENT) to terminate the digestion. The digested kidney tissue suspensions were passed through a mesh with 40 μm pore size to remove the undigested tissues and then washed in PBS. 40% and 70% percoll (17-0891-02, GE Healthcare, Sweden) were used for the gradient centrifugation. The final pellets were resuspended in PBS and then loaded to the flow cytometry for analysis.

After blocking nonspecific Fc binding with anti-mouse CD16/32 (14-0161-85, eBioscience™), fresh renal immune cell suspensions were incubated with anti-mouse CD45 antibody (30-F11, 47-0451-82, APC-eFluor™ 780, eBioscience™) to determine total immune cell numbers. Anti-CD45 antibody-labeled samples were also labeled with the other following antibodies, anti-mouse, F4/80-PE (BM8, 48-4801-82, eBioscience™), CD11c (N418, 17-0114-81, eBioscience™), CD3e (145-2C11, 12-0031-81, eBioscience™), Ly6G/Ly6C (RB6-8C5, 11-5931-82, eBioscience™) and CD19 (eBio1D3 (1D3), 25-0193-81, eBioscience™) antibody to identify neutrophils (CD45Ly6G), macrophages (CD45F4/80), T cells (CD45Cd3e), dendritic cells (CD45CD11c) and B cells (CD45CD19) respectively. Using CD206-FITC (MR5D3, MA5-16870, TheroFisher Scientific) and CD11b (M1/70, 15-0112-82, eBioscience™) antibody, we identified M1 (CD11bCD206) and M2 (CD11bCD206) subtypes in macrophages. Subsequent data acquisition was performed by flow cytometer (CytoFLEXB53015, Beckman).

### RNA analysis

Total RNA was obtained from freshly isolated renal proximal tubules by RNA-iso reagent (TakaRa). Total RNA was reverse-transcribed to cDNA using Reverse Transcription Kit (BGI, Shenzheng, China). The levels of Acat3, Cyp4a31, Cyp4a10, Cyp4a14, Ugt2b34 and Nectin1 were determined by SYBRGreen I Real-time quantitative PCR in a CFX96 real-time RT-PCR detection system (Bio-Rad). PCR amplification was carried out for 42 cycles. The following primer sequences were used: Acat3 (forward: 5’-CTGGAGGCATGGAGAATATGAG-3’, reverse: 5’-CTGTCAGACCATCACAGAGTATG-3’); Cyp4a31 (forward: 5’-CTACCCTGCATAGTCTCTCTCT-3’, reverse: 5’-GCATGACACTTGGACCTTTATTG-3’); Cyp4a10 (forward: 5’-TTCCCTGATGGACGCTCTTTA-3’, reverse: 5’-GCAAACCTGGAAGGGTCAAAC-3’); Cyp4a14 (forward: 5’-TCTGGGTTCTTCCAATGGGC-3’, reverse: 5’-GGACTCGTATATTGCTCCCCG-3’); Ugt2b34 (forward: 5’-TGAAGTGATGGTTCTGAGACCT -3’, reverse: 5’-ACTGCTTTGGCAGCTCATAAAT-3’); Nectin1 (forward: 5’-GACTCCATGTATGGCTTCATCG-3’, reverse: 5’-CACTCGTTTCTCGTAGGGAGG-3’).

## Supplementary information

Supplemental materials

checklist

Supplemental Table 1

Supplemental Table 2

Supplemental Table 3

Supplemental Table 4

Supplemental Table 5

Supplemental Table 6

## Data Availability

The sequencing data have been deposited in the National Center for Biotechnology Gene Expression Omnibus, https://www.ncbi.nlm.nih.gov/geo/ (accession no. GSE197266). Any additional information required to reanalyze the data reported in this paper is available from the lead contact upon reasonable request.
