# Supplementary material for: Single-cell sequencing reveals homogeneity and heterogeneity of the cytopathological mechanisms in different etiology-induced AKI
Source: Cell Death Dis. 2023 May 11;14(5):318. doi: 10.1038/s41419-023-05830-z (PMC10175265; doi:10.1038/s41419-023-05830-z)
Supplement: Supplementary file 1 — Supplemental materials [file 41419_2023_5830_MOESM1_ESM.pdf]

Supplementary Materials for

Single-cell sequencing reveals homogeneity and heterogeneity of the cytopathological mechanisms in different etiology-induced AKI

Zhimin Chen[1,2,3#], Yinshuang Li[1,2,3#], Ying Yuan[1,2,3#], Kunmei Lai[1,2,3], Keng Ye[1,2,3], Yujiao Lin[1,2,3], Ruilong Lan[4], Hong Chen[5], Yanfang Xu[1,2,3,4]*

[#] These authors contributed equally
* Correspondence to: xuyanfang99@hotmail.com (Y.X.)

This file includes:

Supplementary Figs S1 to S19
Captions for Supplementary Tables S1 to S6

Other Supplementary Materials for this manuscript include the following as an Excel file:

Supplementary Tables S1 to S6

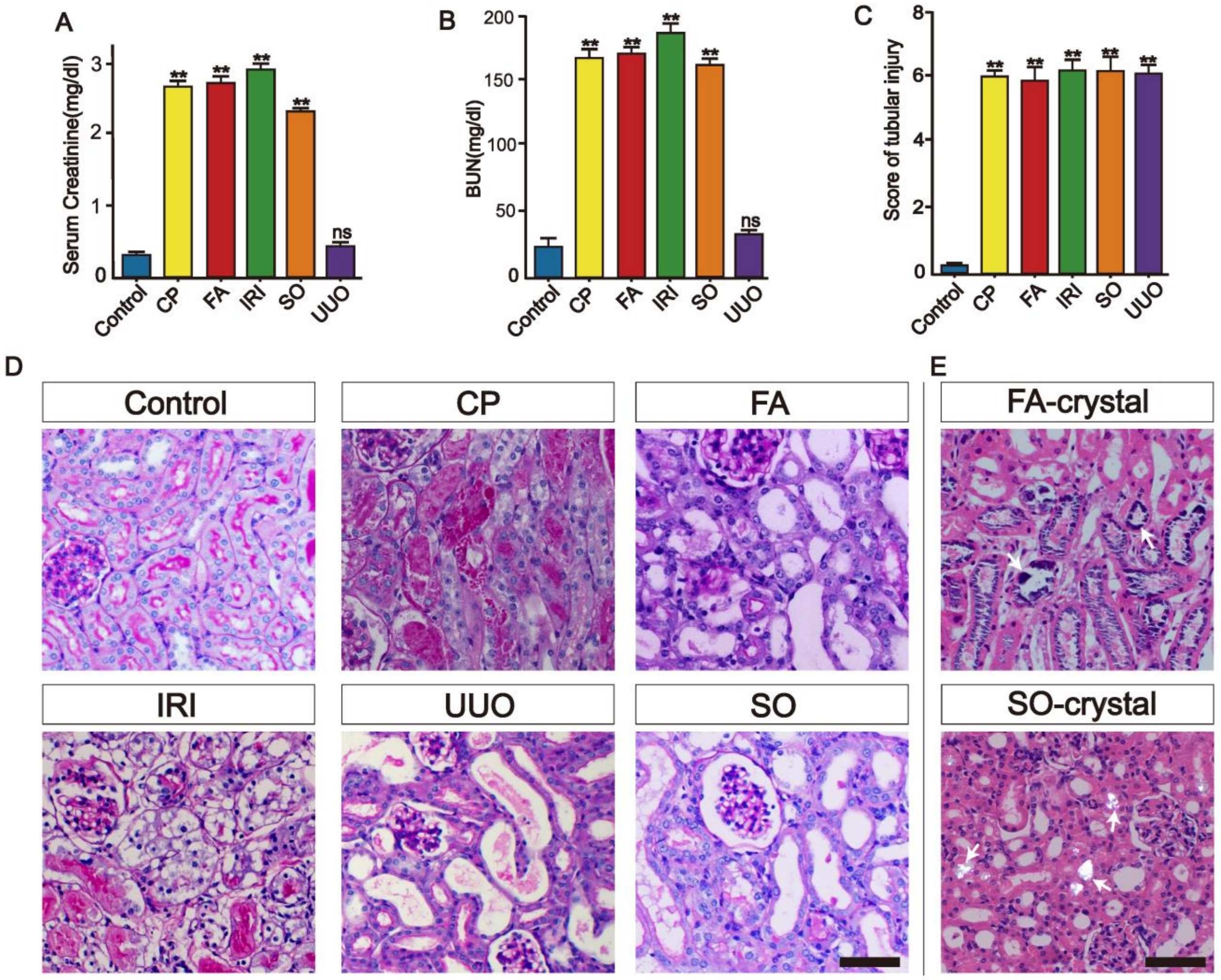

Supplemental Fig. S1. Renal histopathological and serum marker changes in different AKI mice.

(A and B) Levels of creatinine (Scr) and blood urea nitrogen (BUN) of the Control-group mice and AKI-mice induced by cisplatin (CP), folic acid (FA), sodium oxalate (SO), ischemia reperfusion injury (IRI), and unilateral ureteral obstruction (UUO). Since UUO mice are unilateral ureteral obstruction, it is normal that Scr and BUN levels do not change significantly in the early stages of AKI; means±SEM. One-way ANOVA test, n = 3, $^{**}P<0.01$ vs. Control group, *ns*: not significant.

(C) Histologic renal injury scores of the Control-group mice and AKI-mice induced by different etiology. The scores were obtained by counting the percentage of tubules that displayed tubular brush loss, necrosis and tubular dilation and cast formation in panel **(D).** At least 10 fields were counted on each section; means±SEM. One-way ANOVA test, n = 3, $^{**}P<0.01$ vs. Control group, *ns*: not significant.

(D) Representative periodic acid-Schiff (PAS) staining of kidney sections from Control-group mice and AKI-mice. Scale bars= 100 μm.

(E) The formation of crystals in FA and SO was confirmed by hematoxylin-eosin (HE) staining under polarized light microscopy. Scale bars= 100 μm.

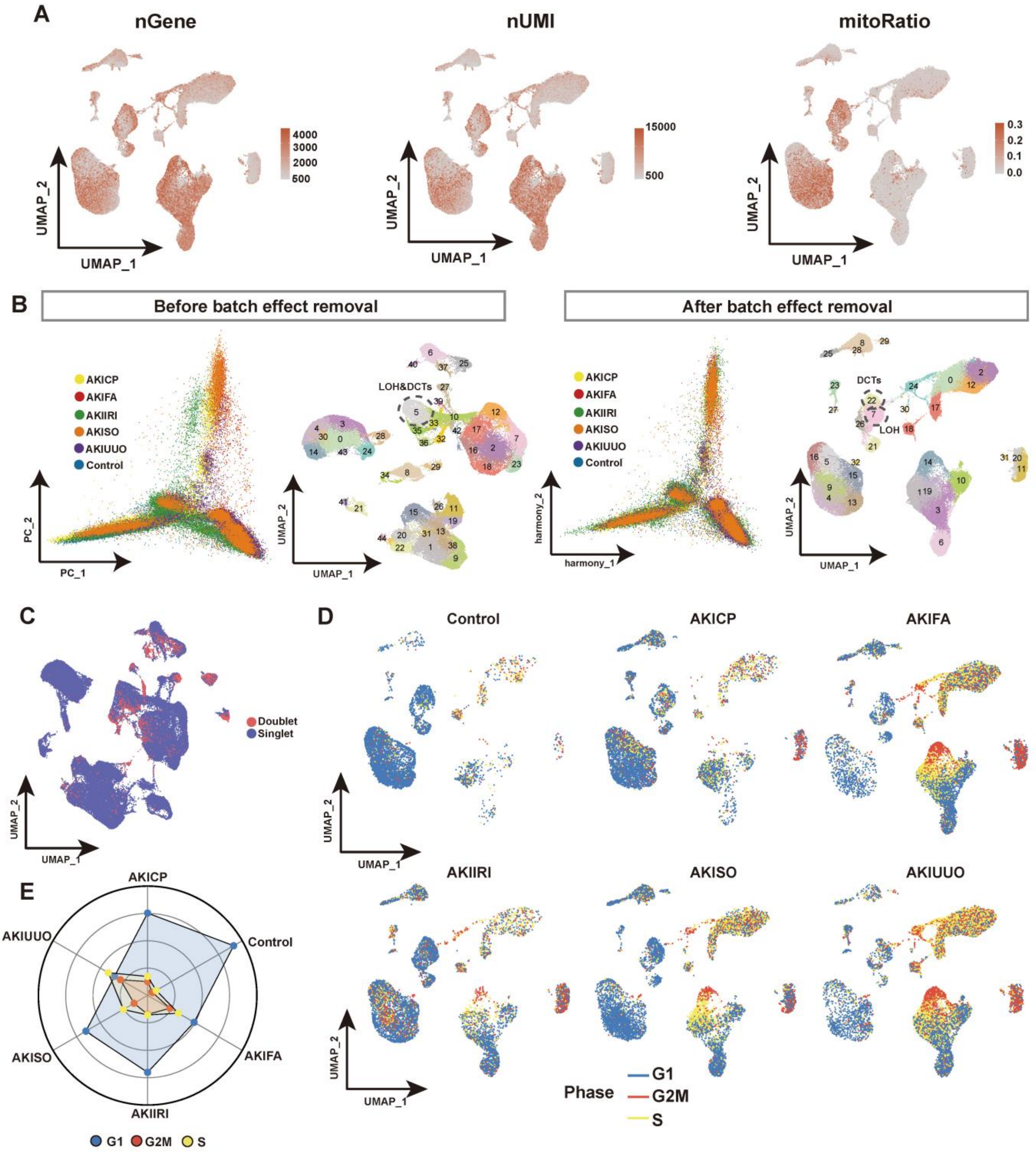

Supplemental Fig. S2. Quality control and remove of batch effects of scRNA data.

(A) The gene count, UMI count and mitochondrial ratio were mapped to the uniform manifold approximation and projection (UMAP) graph after data quality control. Because tubular cells in the kidney are highly metabolically active, therefore exhibit a higher mitochondrial ratio compared to other cell types. Due to the proportion of proximal tubules more than 70% in Control samples, they exhibit a higher mitochondrial proportion.

(B) The principal component analysis (PCA) plots of the combined samples before and after removal of the batch effect, the uniform manifold approximation and projection (UMAP) nonlinear dimensionality reduction plots, and the cluster-UMAP plots. After removing the batch effect, the differences between cells were more obvious, and distal convoluted tubules (DCTs) and loop of Henle (LOH) were separated. In contrast, before the removal of the batch effect, DCTs and LOHs could not be separated even the resolution was substantially adjusted. For this dataset, the removal of the batch effect is beneficial for the subsequent analysis. *n* = 2 each group.

(C) A total of 9075 doublets were identified by the DoubletFinder package, and these were removed in subsequent analyses.

(D and E) After the onset of AKI induced by different etiologies, renal cells show different degrees of cell cycle arrest. Since cell cycle arrest is one of the disease characteristics of AKI, we did not regress out the effect of cell cycle on the dataset in the subsequent analysis. *n* = 2 each group.

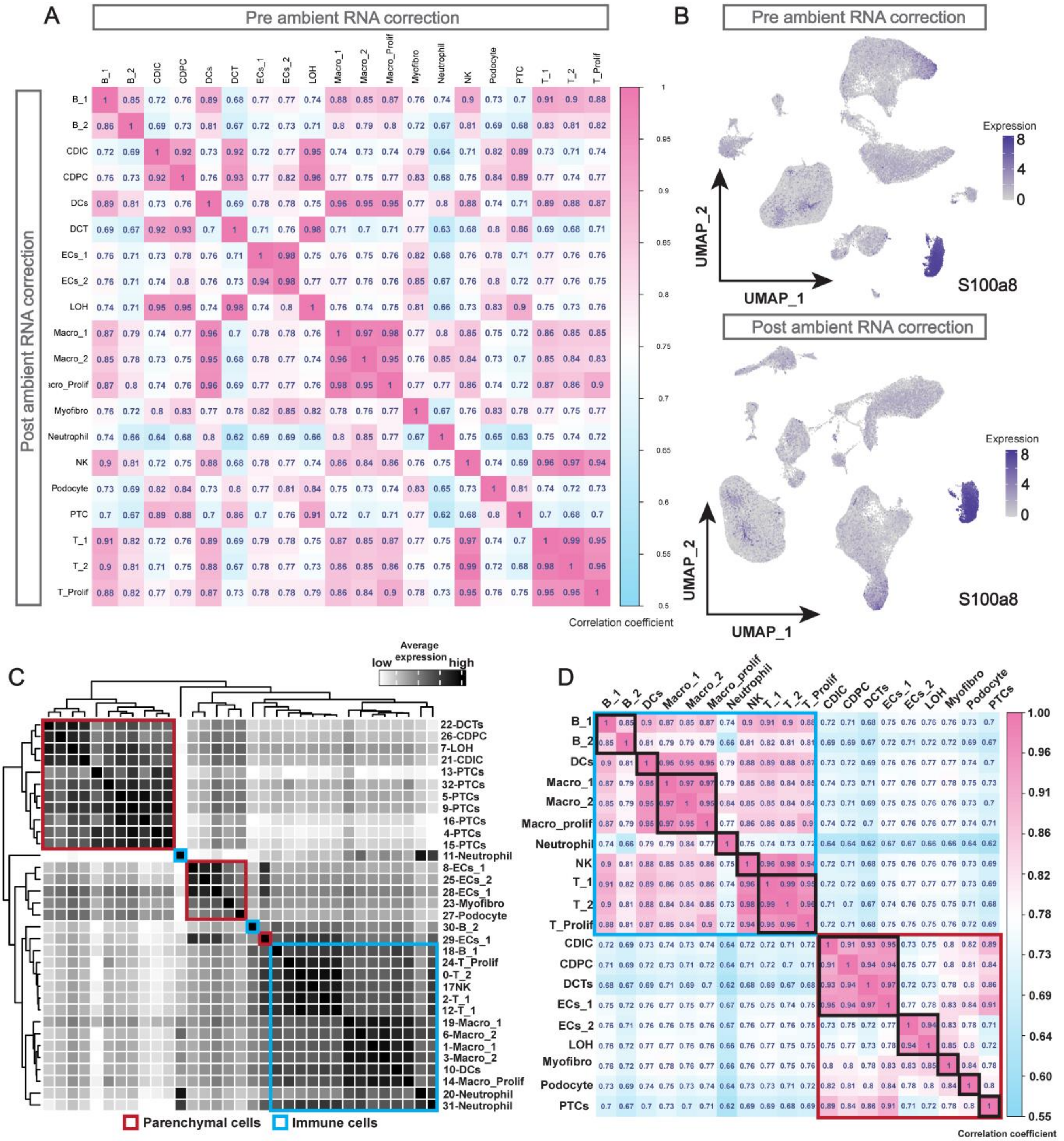

Supplemental Fig. S3. SoupX package to correct potential ambient RNA contamination and the accuracy verification of cell type identification in the dataset.

(A) The correlation heatmap of each celltypes before and after SoupX correction.

(B) Expression of *S100a8* before and after SoupX correction.

(C) Clustering heatmap of the average expression for each celltype, with immune cells and parenchymal cells clustered into separate groups.

(D) The correlation heat map of the average expression of each celltype, with poor correlation between immune cells and parenchymal cells.

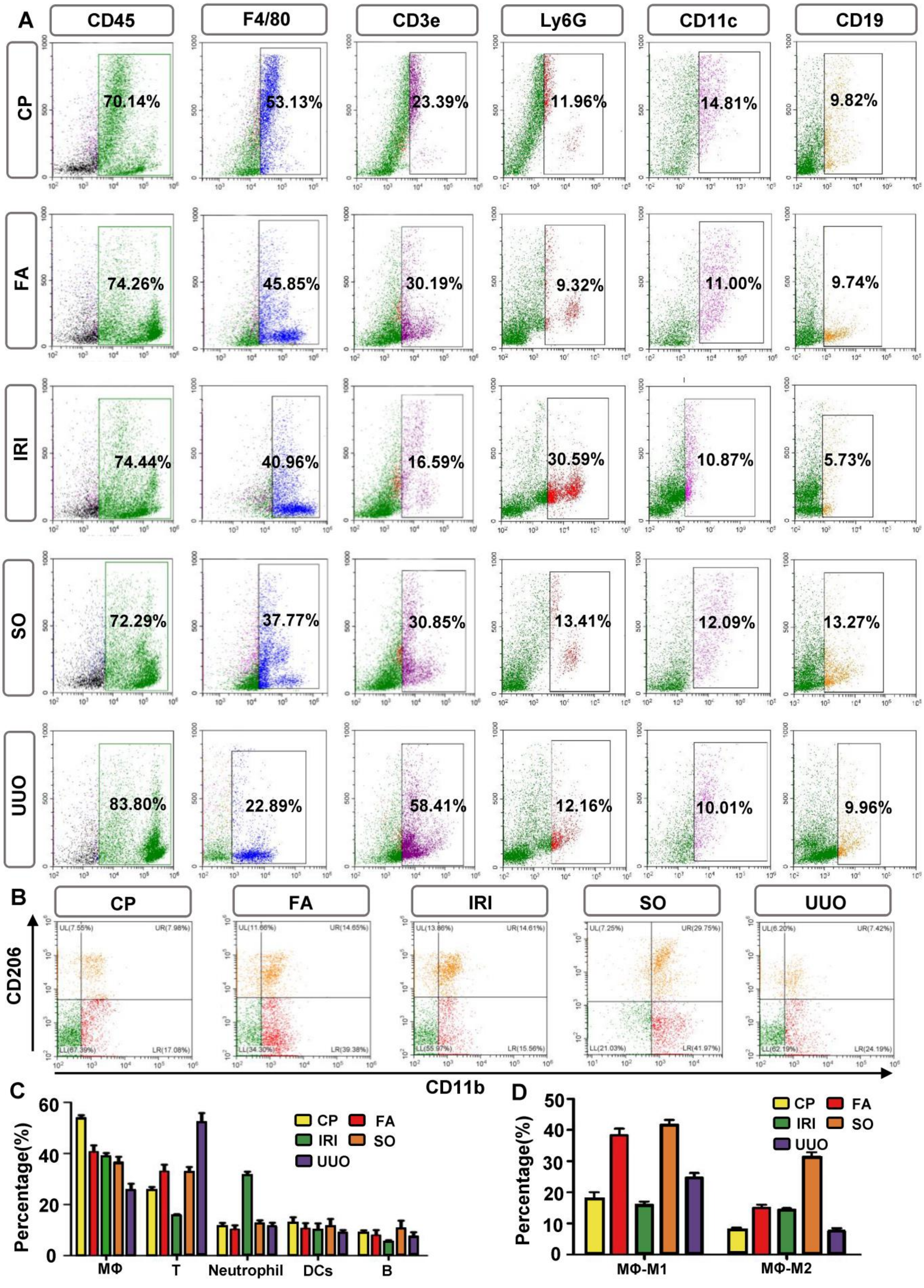

Supplemental Fig. S4. Flow cytometry analysis of the major renal immune cell subtypes in different AKI model.

(A) Immune cell infiltration in the kidneys were further identified in CK, FA, IRI, SO, and UUO-induced AKI by Flow cytometry. Immune cells were distinguished by CD45 (Ptprc), and subsequently identified by F4/80 (Adgre1) for macrophages (MΦ), CD3e for T cells, Ly6G for neutrophils, CD11c (Itgax) for dendritic cells (DCs), and CD19 for B cells. n = 3.

(B) M1 ($CD11b^{+}CD206^{-}$) and M2 ($CD11b^{+}CD206^{+}$) type macrophages, n = 3.

(C) Cell proportions of various immune cell subtypes in panel (A). n = 3.

(D) Cell proportions of M1 and M2 macrophages in panel (B). M1 and M2 type macrophages correspond to Macro_1 and Macro_2. n = 3.

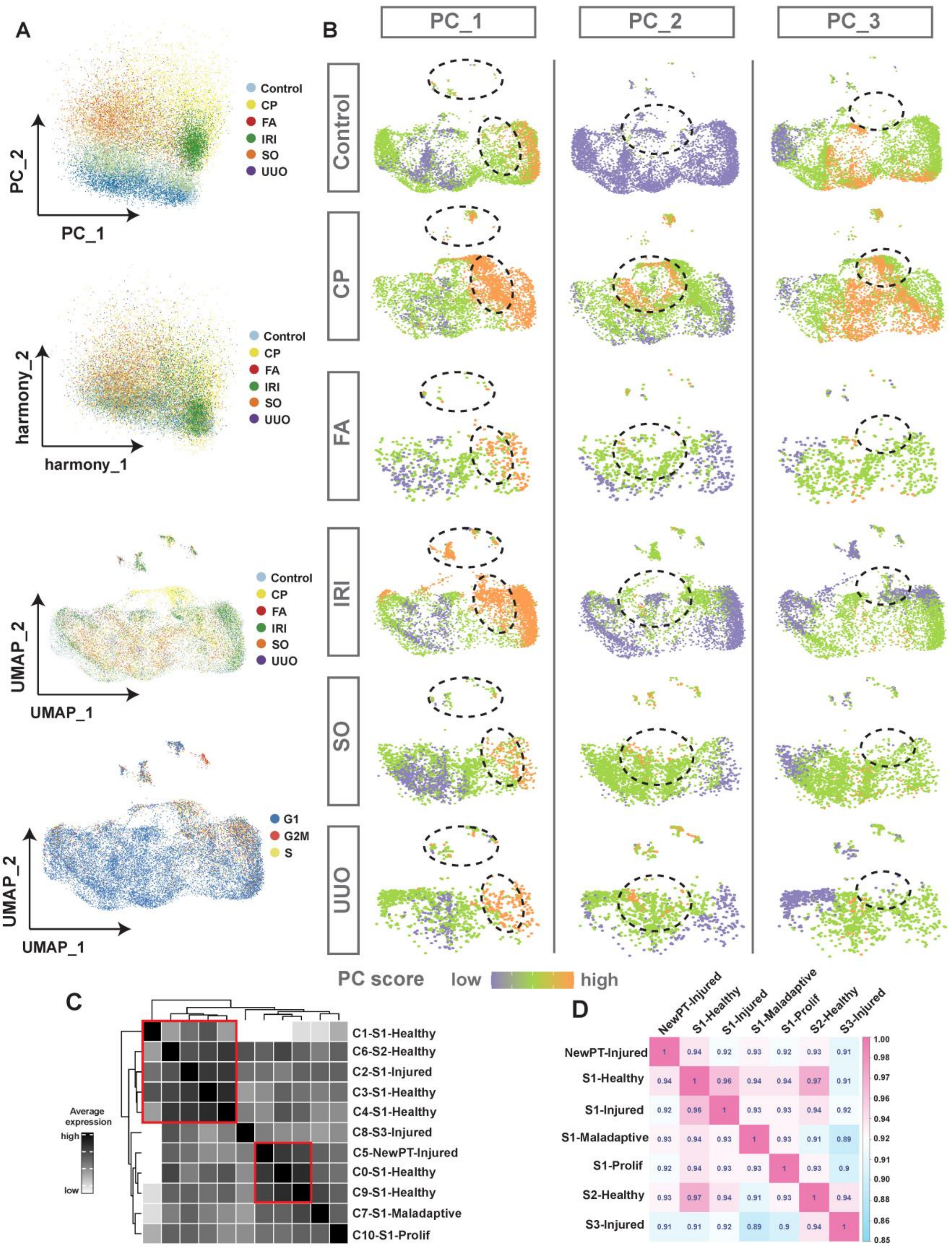

Supplemental Fig. S5. Reclustering of PTCs and identification of AKI-specific PTCs.

(A) Principal component analysis (PCA) plot of PTCs-samples before and after removal of batch effect by harmony algorithm. And distribution of potential batch effect factors on uniform manifold approximation and projection (UMAP) plots after removal of batch effects. *n* = 2 each group.

(B) Scoring of the top three components of the principal component analysis on UMAP. Displayed by model, it can be found that the PTCs of the injury state are the main factor causing the differences between the models.

(C) The clustered heatmap of the average expression of the 11 clusters identified by PTCs after re-clustering, combined with the spatial distribution of clusters after UMAP dimensionality reduction, two large subgroups could be distinguished.

(D) Correlation heat map of the average expression of the 7 PTCs. Cells within the PTCs were highly correlated with a minimum correlation coefficient of 0.89.

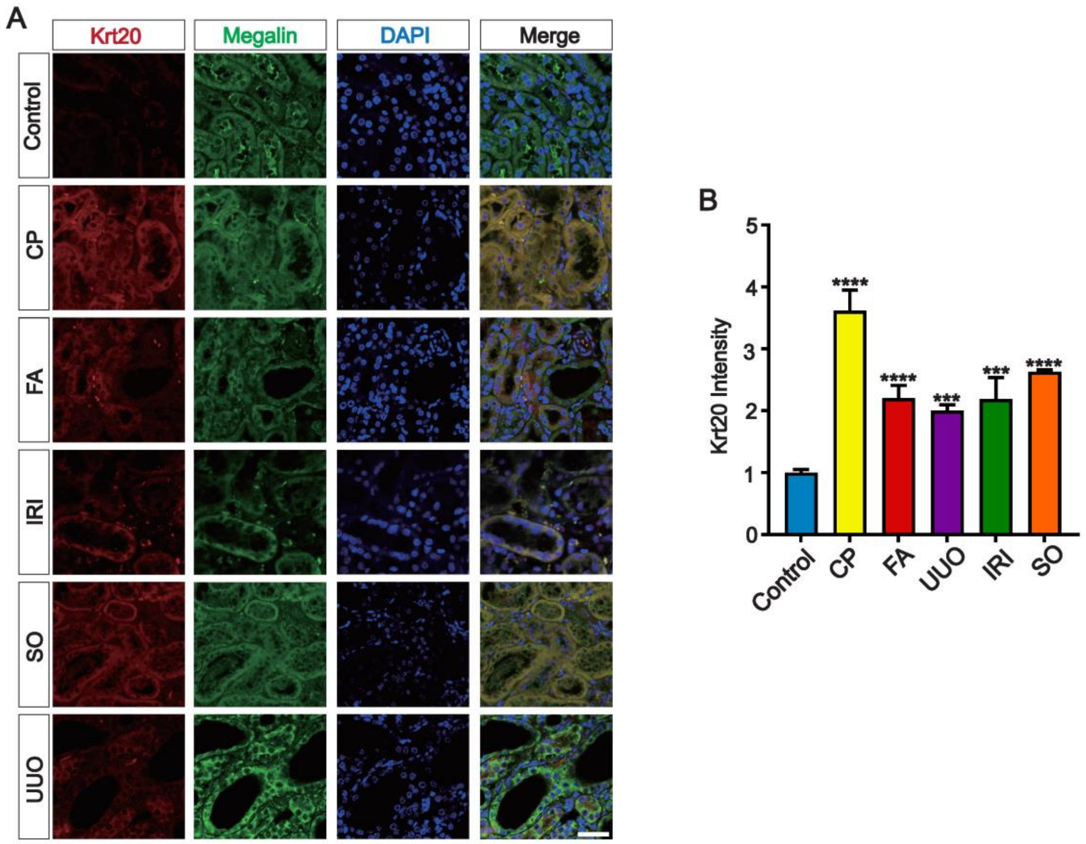

Supplemental Fig. S6. Expression of Krt20 in PTCs.

(A) Co-staining of sections from Control, cisplatin (CP), folic acid (FA), sodium oxalate (SO), ischemia reperfusion injury (IRI), and unilateral ureteral obstruction (UUO) kidneys for Krt20 (red) and Megalin (green) . Scale bars = 100μM. Krt20 is a biomarker of injury state PTCs (InjuredNewPT).

(B) The quantitative analysis of Krt20 fluorescence intensity; means ± SEM. One-way ANOVA test, $n = 3$, ***$P < 0.001$, ****$P < 0.0001$ vs. Control group.

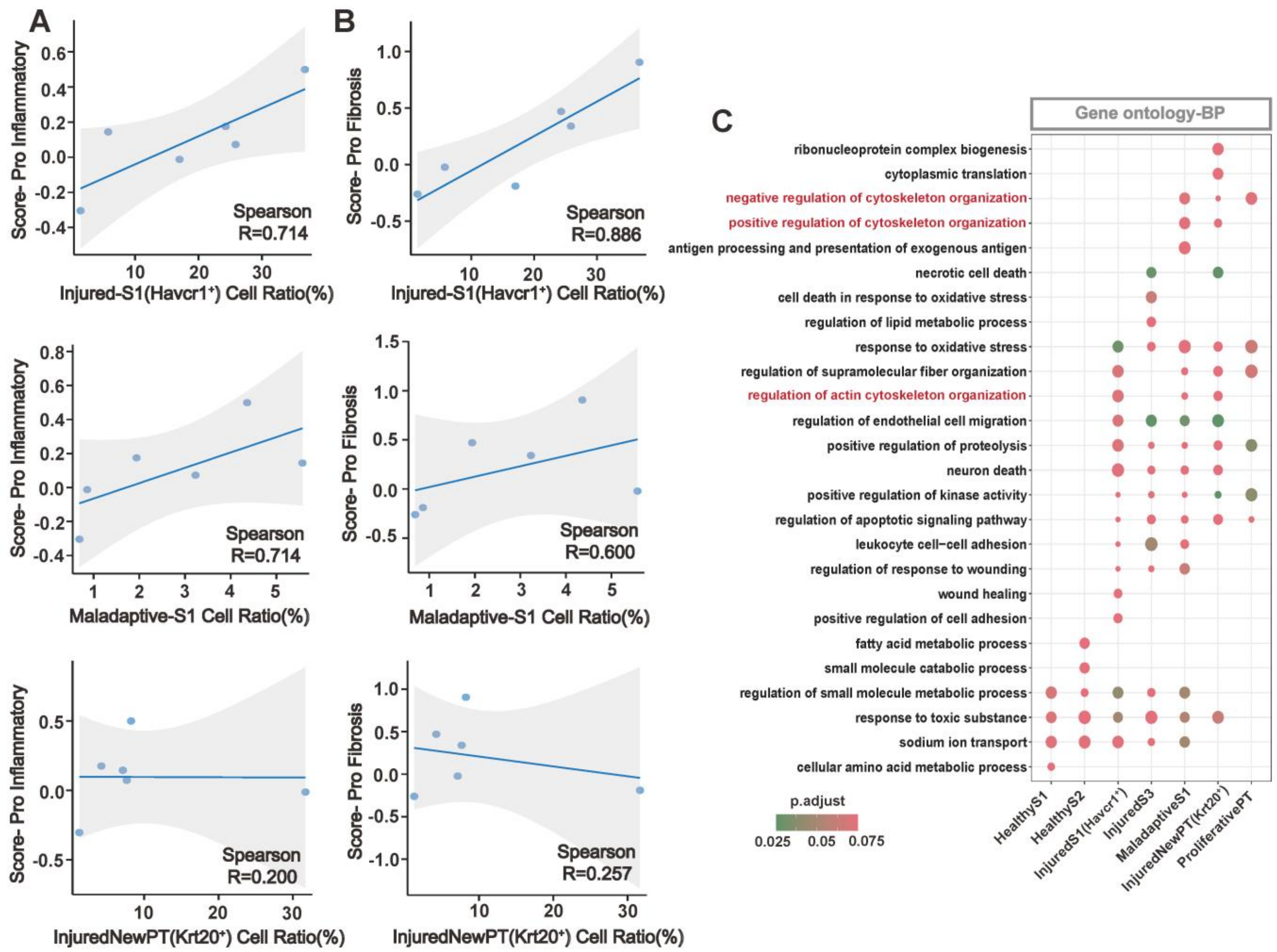

Supplemental Fig. S7. Correlation analysis of the ratio of injured cells with pro-inflammatory and pro-fibrotic scores and gene ontology analysis of PTC subclusters.

(A and B) The cell proportions of injury state PTCs in different models: InjuredS1 (*Havcr1*$^{+}$), Maladaptive-S1, and InjuredNewPT (*Krt20*$^{+}$) were correlated with the pro-inflammatory (A) and pro-fibrotic (B) scores of these models, and it was found that the cell proportions of InjuredS1 (*Havcr1*$^{+}$) and Maladaptive-S1 were positively correlated with the pro-inflammatory and pro-fibrotic scores of the models, while InjuredNewPT (*Krt20*$^{+}$) showed relatively weak correlation.

(C) Biological processes involved in 7 different states of PTCs in gene ontology analysis as a complement to **Figure 3A**. Positive and negative cytoskeletal reorganization events and inflammatory processes associated with cell death are common features of injured PTCs.

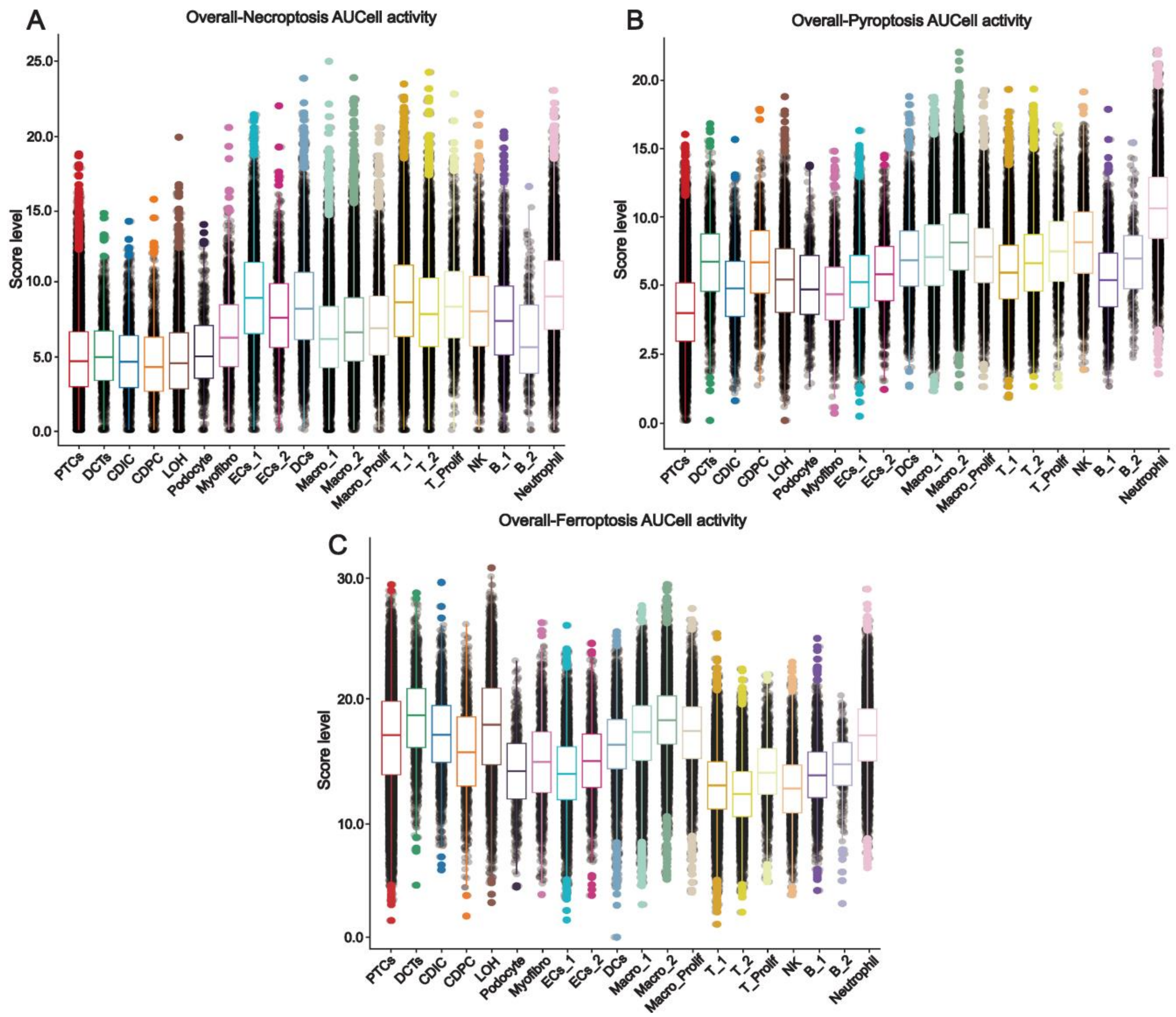

Supplemental Fig. S8. Overall cell death score for all cell types.

(A-C) Necroptosis (A), Pyroptosis (B), and Ferroptosis (C) scores for all cell types, Supplement to **Figure3A-C**. Necroptosis had high pathway activity in most parenchymal and immune cells, more significantly in neutrophils, endothelial cells, T cells, dendritic cells, and macrophages. Pyroptosis had high pathway activity in specific immune cells, especially in macrophages, NK cells, and neutrophils. Ferroptosis showed high pathway activity in different segments of renal tubular cells, macrophages and neutrophils.

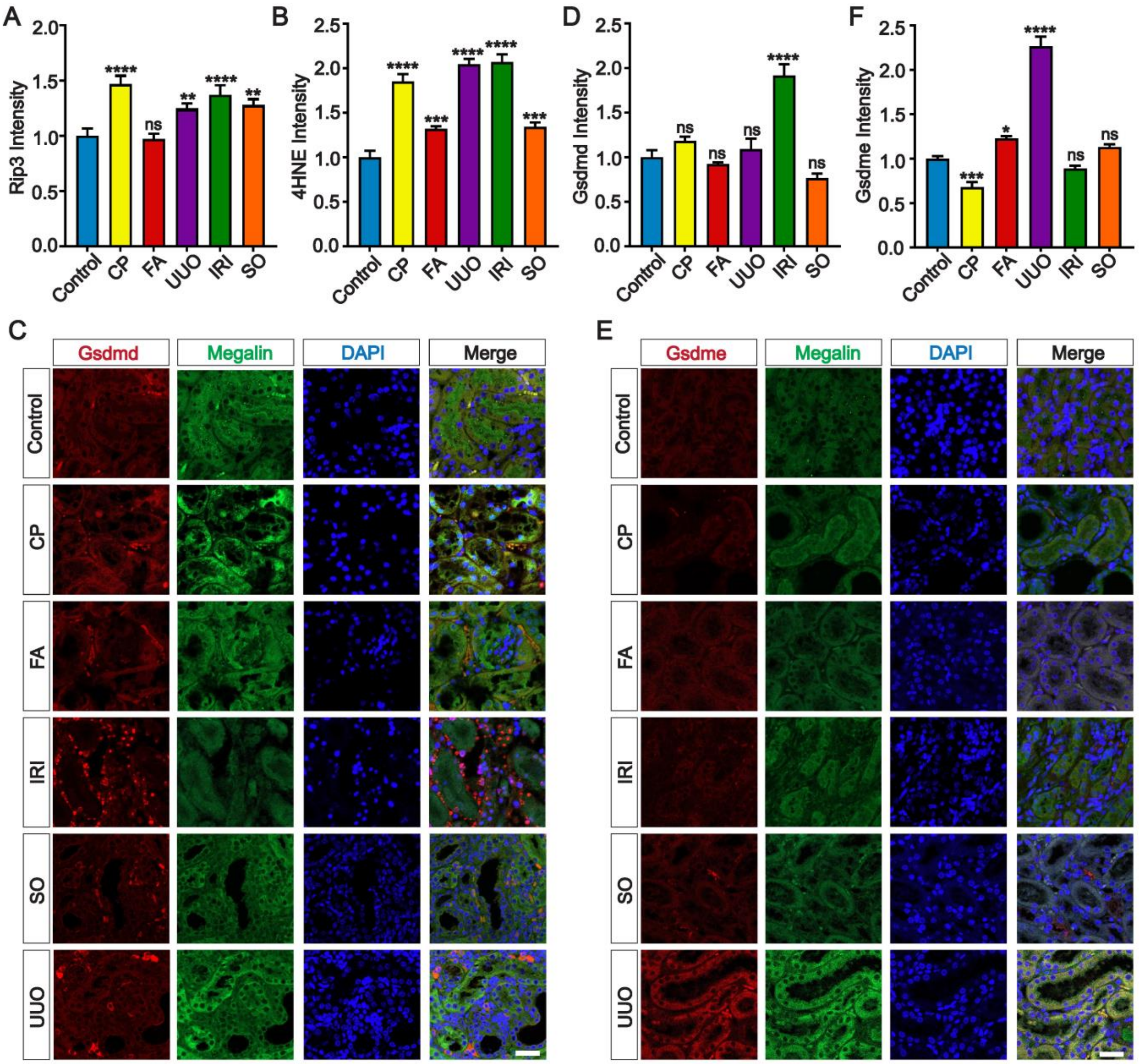

Supplemental Fig. S9. Expression of key cell death markers in different models of PTCs.

(A) The quantitative analysis of Rip3 fluorescence intensity, as a complement to Figure3G; means ± SEM. One-way ANOVA test, $n = 3$, ***$P < 0.001$, ****$P < 0.0001$ vs. Control group, *ns*: not significant.

(B) The quantitative analysis of 4HNE fluorescence intensity, as a complement to Figure3H; means ± SEM. One-way ANOVA test, $n = 3$, ***$P < 0.001$, ****$P < 0.0001$ vs. Control group.

(C and E) Co-staining of sections from Control, cisplatin (CP), folic acid (FA), sodium oxalate (SO), ischemia reperfusion injury (IRI), and unilateral ureteral obstruction (UUO) kidneys for Gsdmd (C, red), Gsdme (E, red) and Megalin (green). Scale bars = 100μM. Gsdmd and Gsdme are involved in Pyroptosis. Gsdmd as a novel biomarker for PTCs in IRI models and Gsdme-mediated Pyroptosis in UUO models have been demonstrated.

(D and F) The quantitative analysis of Gsdmd **(D)** and Gsdme **(F)** fluorescence intensity; means ± SEM. One-way ANOVA test, $n = 3$, *$P < 0.05$, ***$P < 0.001$, ****$P < 0.001$ vs. Control group, *ns*: not significant.

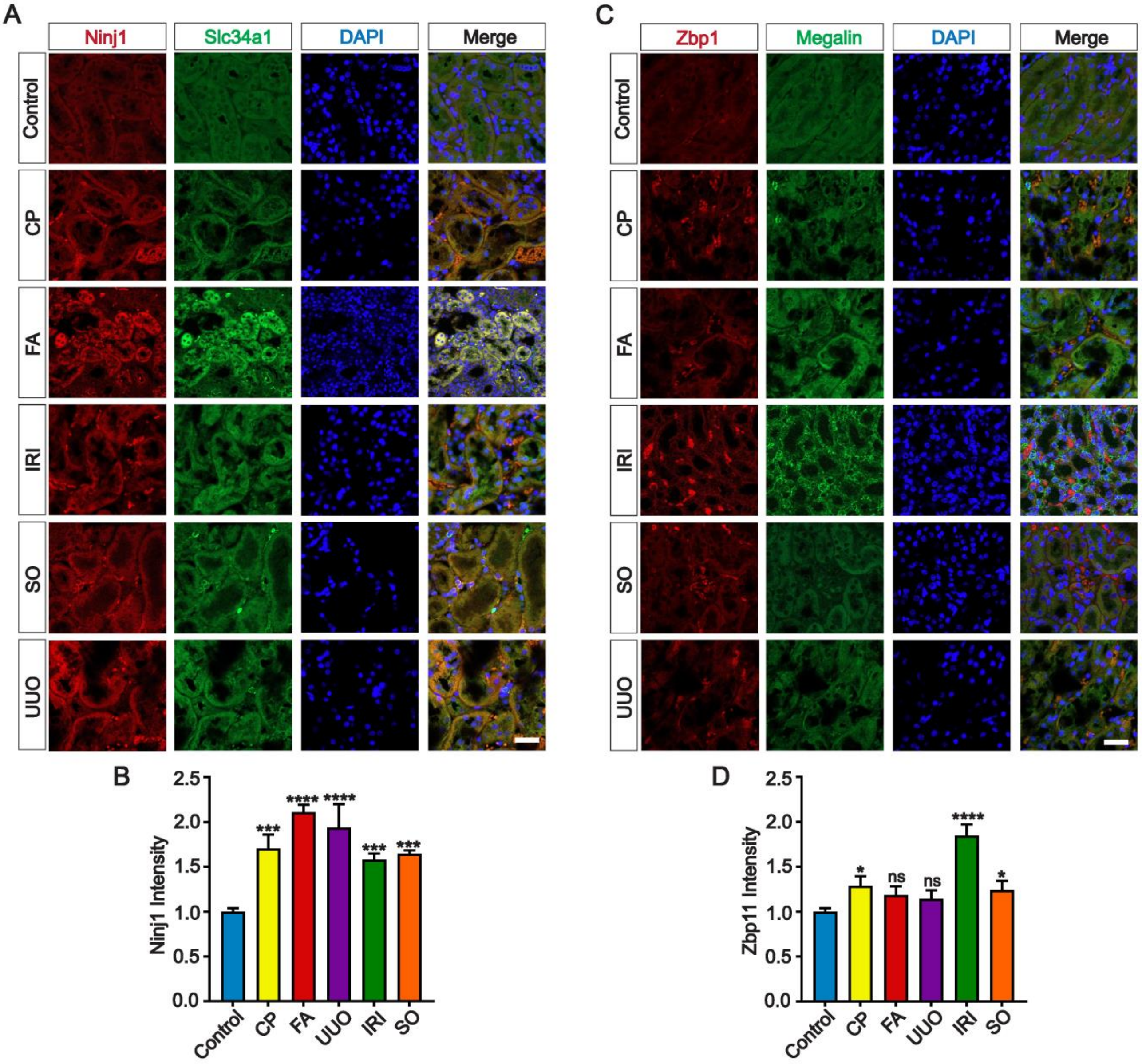

Supplemental Fig. S10. Expression of Ninj1 and Zbp1 in different models of PTCs.

(A and C) Co-staining of sections from Control, cisplatin (CP), folic acid (FA), sodium oxalate (SO), ischemia reperfusion injury (IRI), and unilateral ureteral obstruction (UUO) kidneys for Ninj1 **(A, red),** Zbp1 **(C, red)** and Megalin (green). Scale bars = 100μM. Ninj1 and Zbp1 were demonstrated to be involved in various cell deaths, but have not been extensively studied in AKI. Ninj1 expressed in renal tubular cells and immune cells, while Zbp1 expressed in interstitial immune cells.

(B and D) The quantitative analysis of Ninj1 **(B)** and Zbp1 **(D)** fluorescence intensity; means±SEM. One-way ANOVA test, $n = 3$, *$P < 0.05$, ***$P < 0.001$, ****$P < 0.001$ vs. Control group, *ns*: not significant.

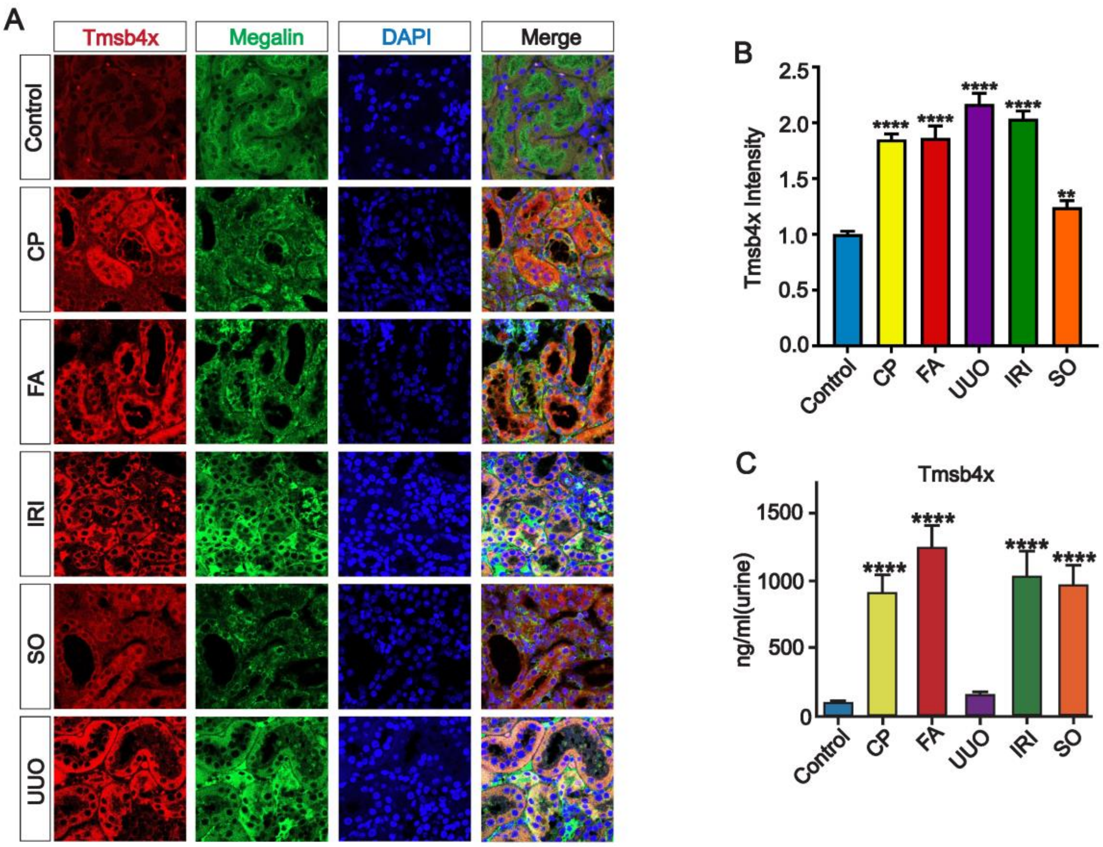

Supplemental Fig. S11. Expression of Tmsb4x in different models of PTCs and detection the level of Tmsb4x in urine.

(A) Co-staining of sections from Control, cisplatin (CP), folic acid (FA), sodium oxalate (SO), ischemia reperfusion injury (IRI), and unilateral ureteral obstruction (UUO) kidneys for Tmsb4x **(A, red)** and Megalin (green). Scale bars = 100μM. Tmsb4x was associated with cellfate1 differentiation in monocle analysis of PTCs and combined with gene enrichment analysis and previous relevant studies, Tmsb4x appeared to affect the transition of PTCs to cellfate1 outcome by participating in the remodeling of the PTCs cytoskeleton.

(B) The quantitative analysis of Tmsb4x fluorescence intensity; means±SEM. One-way ANOVA test, $n = 3$, $^{**}P < 0.01$, $^{****}P < 0.001$ vs. Control group.

(C) The level of Tmsb4x detected in urine of mice by enzyme linked immunosorbent assay (ELISA). means±SEM. One-way ANOVA test, $n = 6$, $^{****}P < 0.001$ vs. Control group. Notably, only one kidney in UUO model is injured since the mice cannot survive with both ureteral ligation, thus the level of Tmsb4x are closely to Control-group**.**

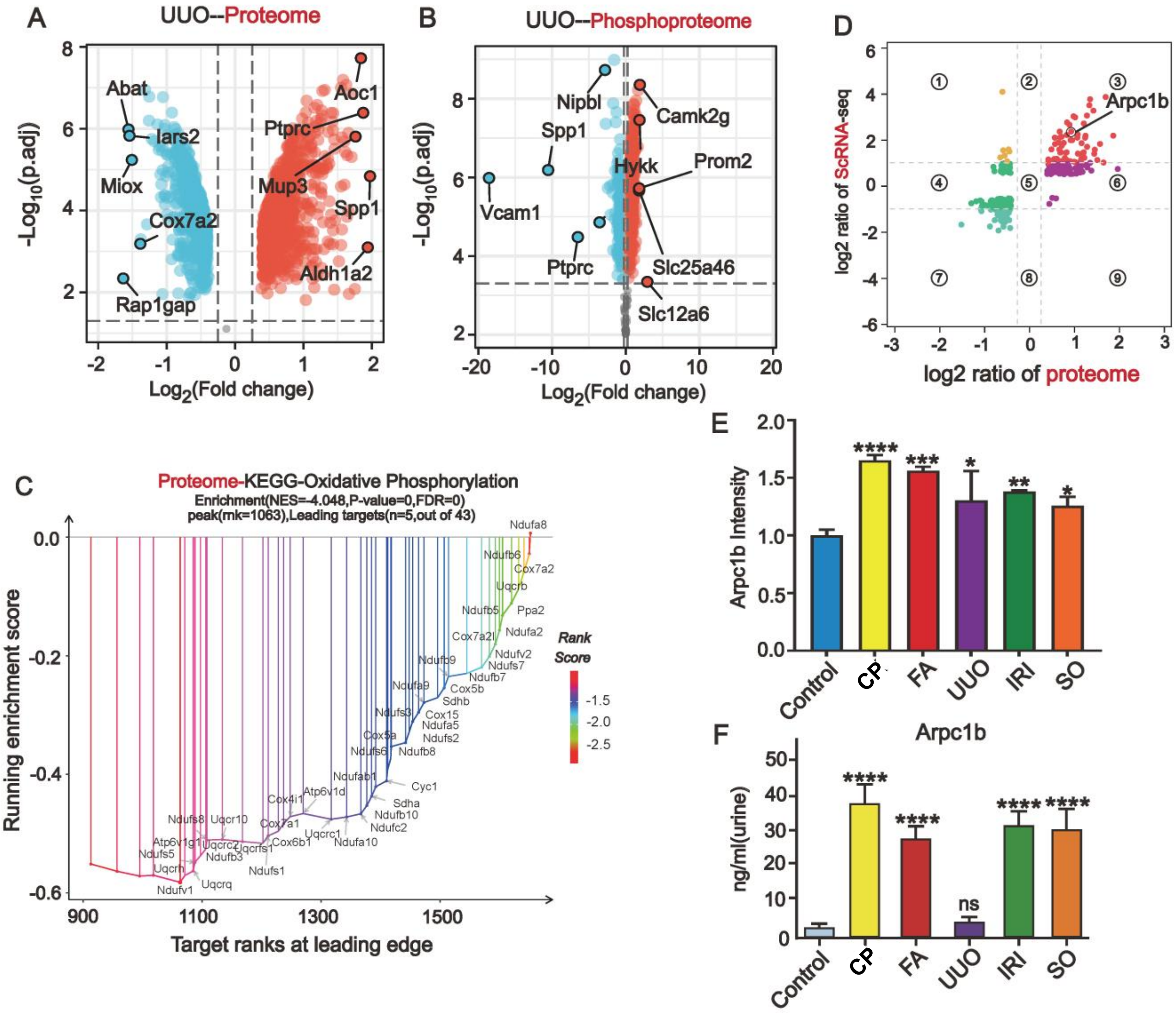

Supplemental Fig. S12. Identification of differentially expressed proteins and phosphorylated proteins in AKI by mass spectrometry.

(A and B) Volcano plots of differentially expressed proteins in UUO-AKI proteomics **(A)** and UUO-AKI phosphoproteomics **(B)** , highlighting the five proteins with the greatest degree of Log2 Fold change among the up- and down-regulated proteins.

(C) GSEA analysis of differentially expressed proteins in the proteomics, and the most significantly enriched pathway in the KEGG was the oxidative phosphorylation pathway.

(D) Cytoskeleton reorganization-related protein Arpc1b is simultaneously highly expressed at the transcriptional and translational levels.

(E) The quantitative analysis of Arpc1b fluorescence intensity, as a complement to **Figure4J**; means±SEM. One-way ANOVA test, n = 3, *P< 0.05, **P< 0.01, ***P< 0.001, ****P< 0.0001 vs. Control group, *ns*: not significant.

(F) The level of Arpc1b detected in urine of mice by enzyme linked immunosorbent assay (ELISA). means±SEM. One-way ANOVA test, n = 6, ****P< 0.001 vs. Control group. Notably, only one kidney in UUO model is injured since the mice cannot survive with both ureteral ligation, thus the level of Arpc1b are closely to Control-group**.**

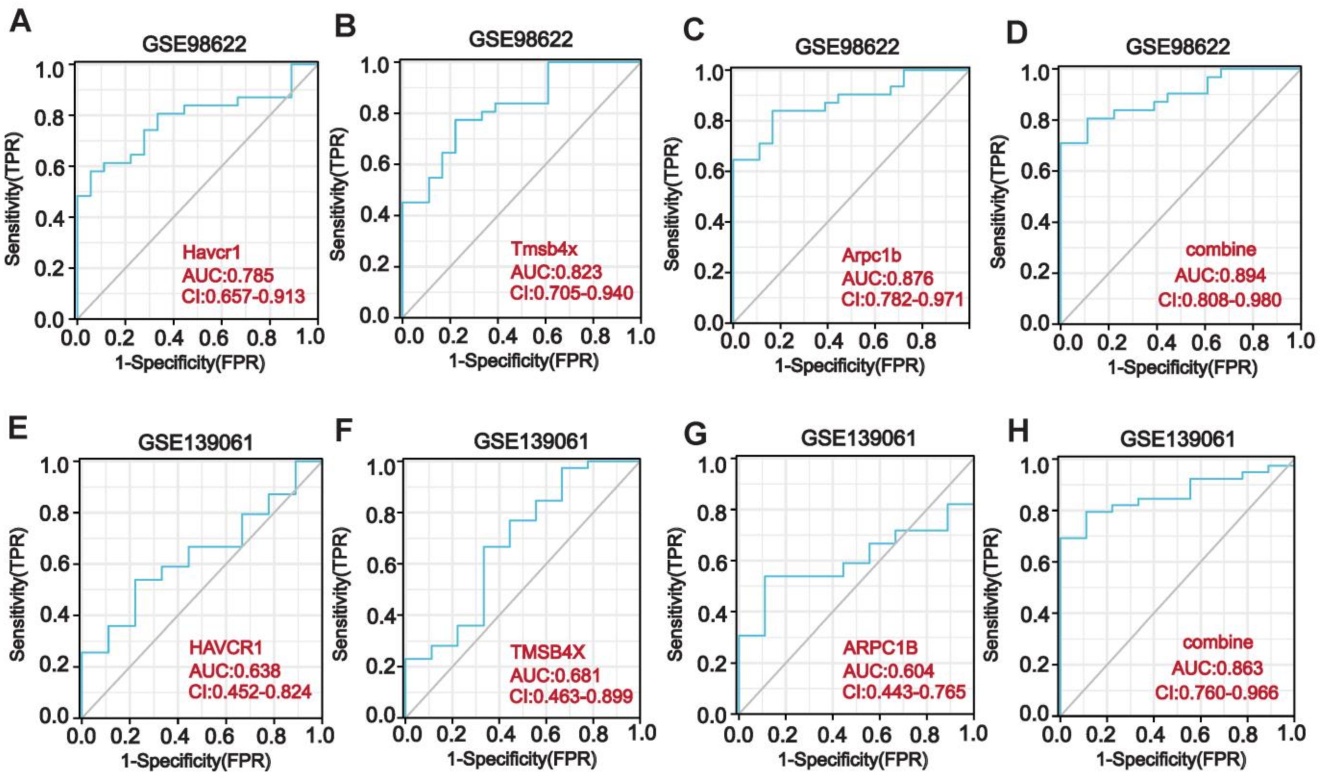

Supplemental Fig. S13. Validation the diagnostic performance of Tmsb4x, Arpc1b through AKI public dataset.

(A-H) Diagnostic efficacy of Havcr1, Arpc1b, Tms4bx and combined genes (*Tmsb4x*, *Arpc1b*, and *Havcr1*) in GSE98622 **(A-D)** and GSE139061 **(E-H)** dataset. The larger value of the area under the curve (AUC) in receiver operating characteristic curve (ROC) indicates the better specificity and sensitivity of the gene for differentiating disease. *Havcr1* (KIM1) is a known marker of kidney injury in AKI.

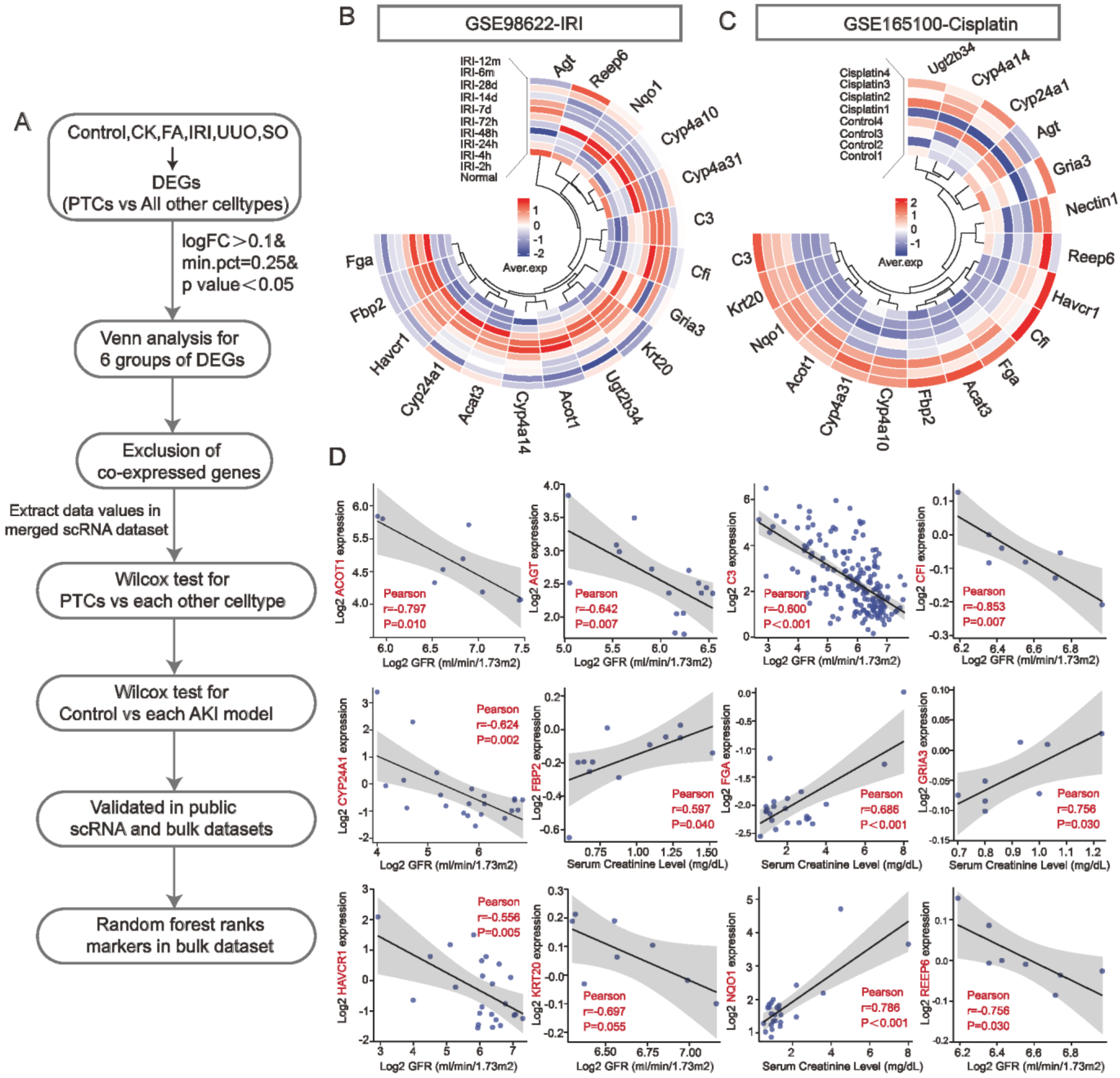

Supplemental Fig. S14. AKI-PTCs specific biomarker screening strategy and validation in bulk dataset.

(A) AKI-PTCs-specific biomarker screening strategy. The aim is to screen for biomarkers of PTCs that are specifically highly expressed in one or more AKIs.

(B) The expression of 18 genes (*Nectin1* is missing in this dataset) was validated in a bulk dataset (Liu J *et.al*, GSE98622) which contains different injury time points of IRI. Most of the genes were expressed starting at IRI-4h and part of them would continue to be highly expressed until 28d.

(C) Expression of 18 genes was verified in the renal tubular dataset of acute cisplatin AKI (Li C *et.al*, GSE165100), and almost all genes were upregulated during AKI.

(D) Exploring the correlation of 18 genes with clinical traits of AKI (GFR: glomerular filtration rate, serum creatinine level) in the Nephroseq database (*Acat3*, *Ugt2b34*, *Nectin1*, *Cyp4a10*, *Cyp4a31*, and *Cyp4a14* are missing in Nephroseq database).

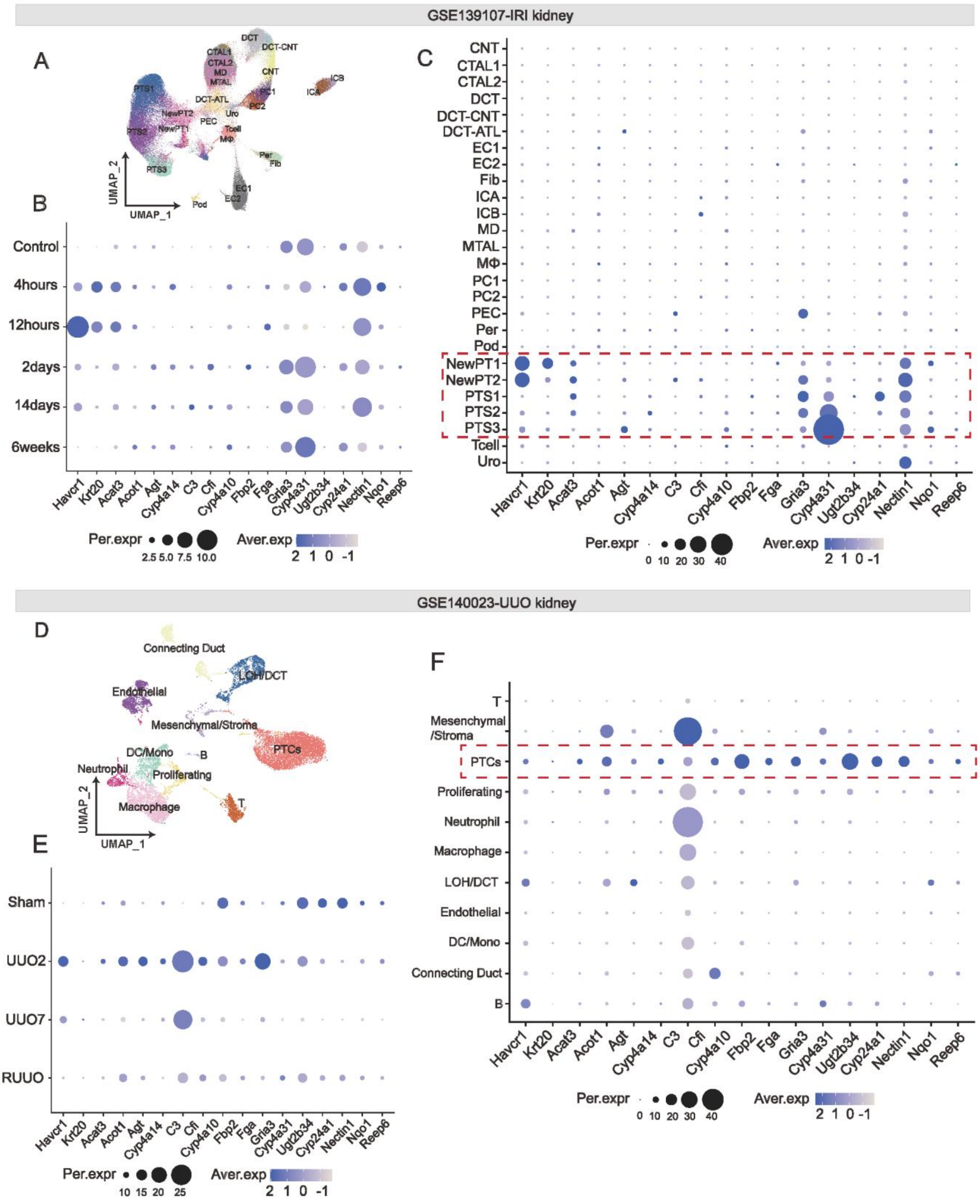

Supplemental Fig. S15. Validation of AKI-PTCs specific biomarkers in two independent scRNA datasets.
(A-C) Expression of 18 genes was validated in single-cell datasets from different injury time points of Ischemia-reperfusion injury (IRI) (A) (Kirita Y *et.al*, GSE139107). Most genes showed elevated expression during IRI 4-12h (B) and restricted expression in PTCs (C) .
(D-F) The expression of 18 genes was validated in a single-cell dataset of unilateral ureteral obstruction (UUO) (D) (Conway BR *et.al*, GSE139107) at different time points of injury. Most genes showed elevated expression during UUO2-7 days (E) and restricted expression to PTCs (F) .

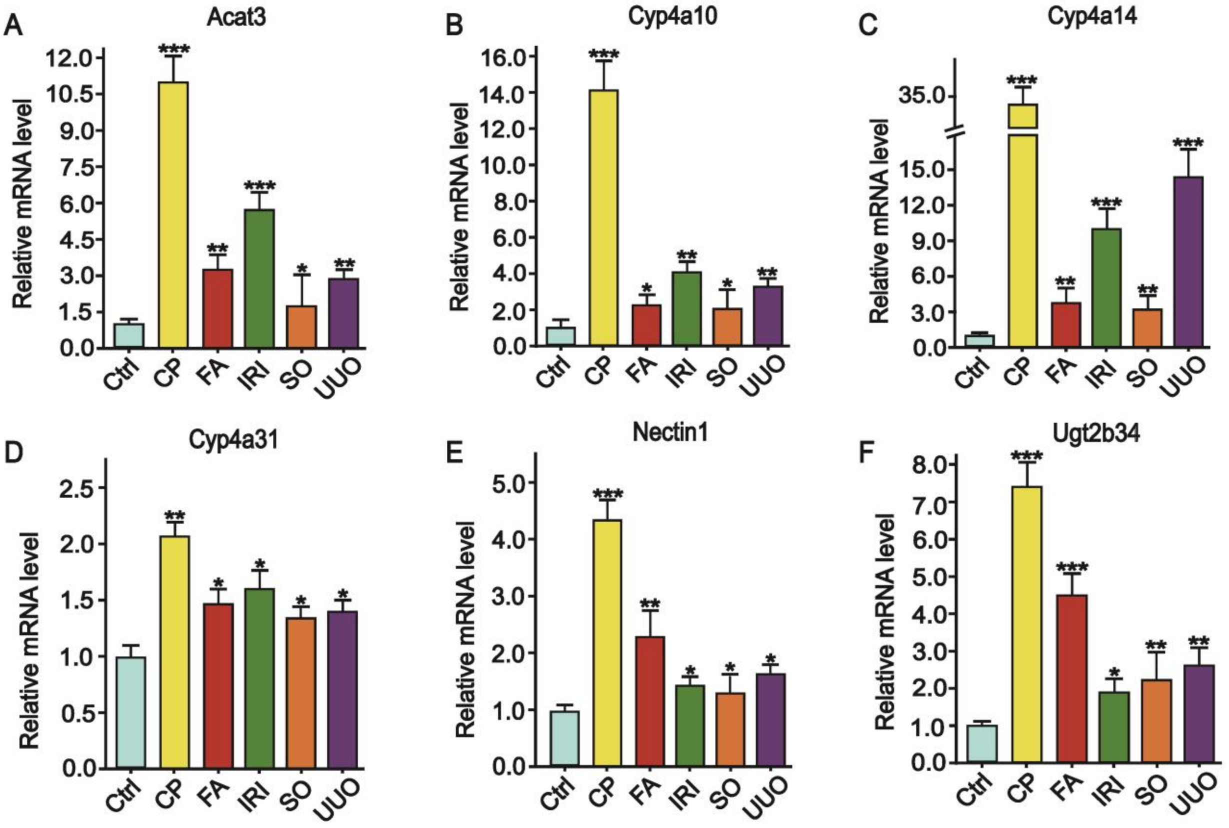

Supplemental Fig. S16. Validation of 6 mouse specific AKI-PTCs biomarkers by Real-time PCR. **(A-F)** Expression of *Acat3***(A),** *Cyp4a10* **(B)**, *Cyp4a14* **(C),** *Cyp4a31* **(D)**, *Nectin1* **(E),** and *Ugt2b34* **(F)** was validated by Real-time PCR ; means±SEM. One-way ANOVA test, $n = 3$, *$P < 0.05$, **$P < 0.01$, ***$P < 0.001$vs. Control group, *ns*: not significant.

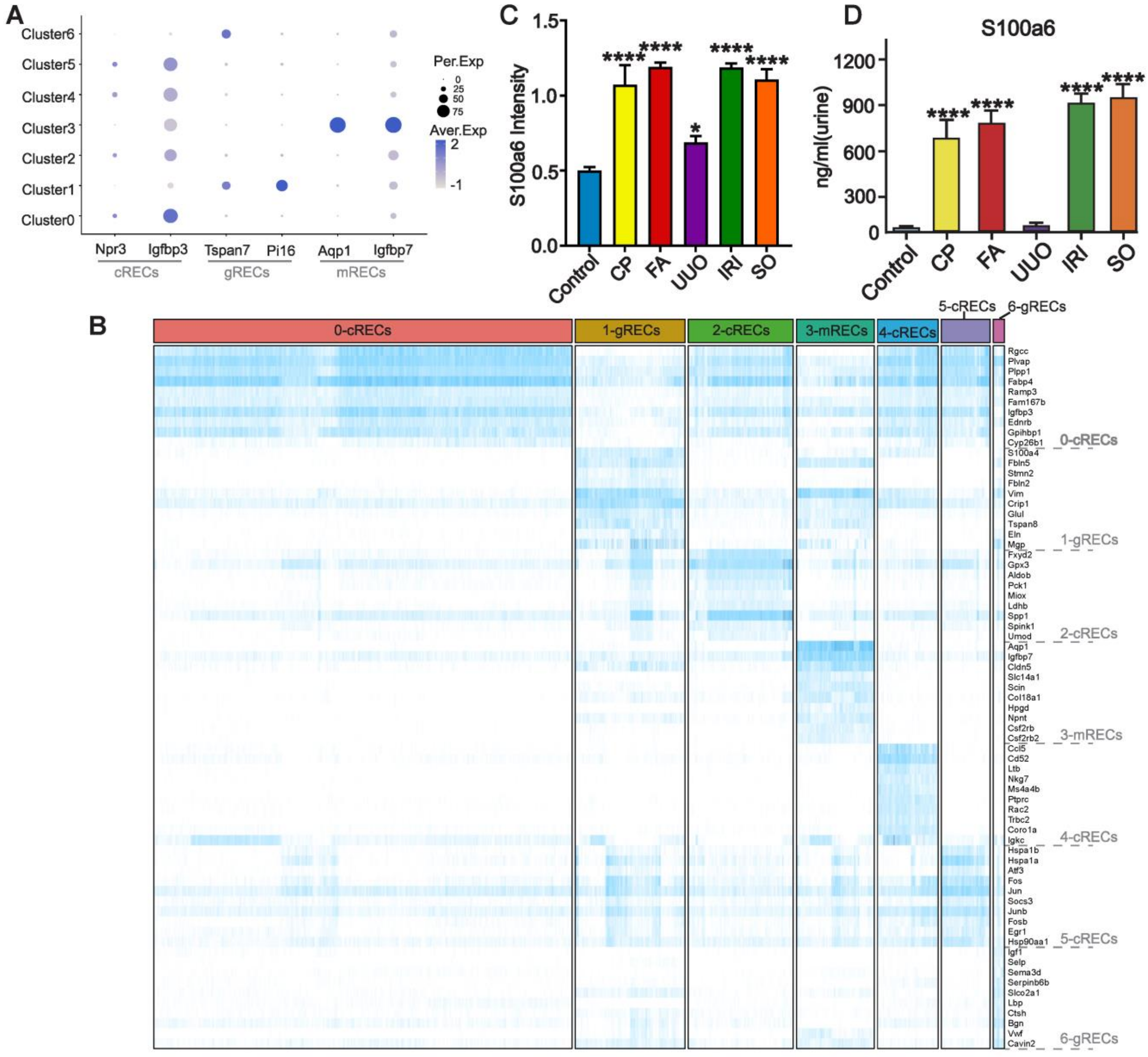

Supplemental Fig. S17. Reclustering of AKI-endothelial cells (ECs) and Top10 gene expression profiles of different endothelial subpopulations.

(A) The clustering heat map of 7 subclusters of EC with samples. AKI models are separated from Control, and the clustering within AKI depends on the variability between different models.

(**B**) Heat map of Top10 differentially expressed genes in three endothelial cell types distributed in 7 clusters.

(**C**) The quantitative analysis of Rip3 fluorescence intensity, as a complement to **Figure6J**; means±SEM. One-way ANOVA test, n = 3, *P< 0.05, ****P< 0.0001 vs. Control group, *ns*: not significant.

(**D**) The level of S100a6 detected in urine of mice by enzyme linked immunosorbent assay (ELISA). means±SEM. One-way ANOVA test, n = 6, ****P< 0.001 vs. Control group. Notably, only one kidney in UUO model is injured since the mice cannot survive with both ureteral ligation, thus the level of S100a6 are closely to Control-group**.**

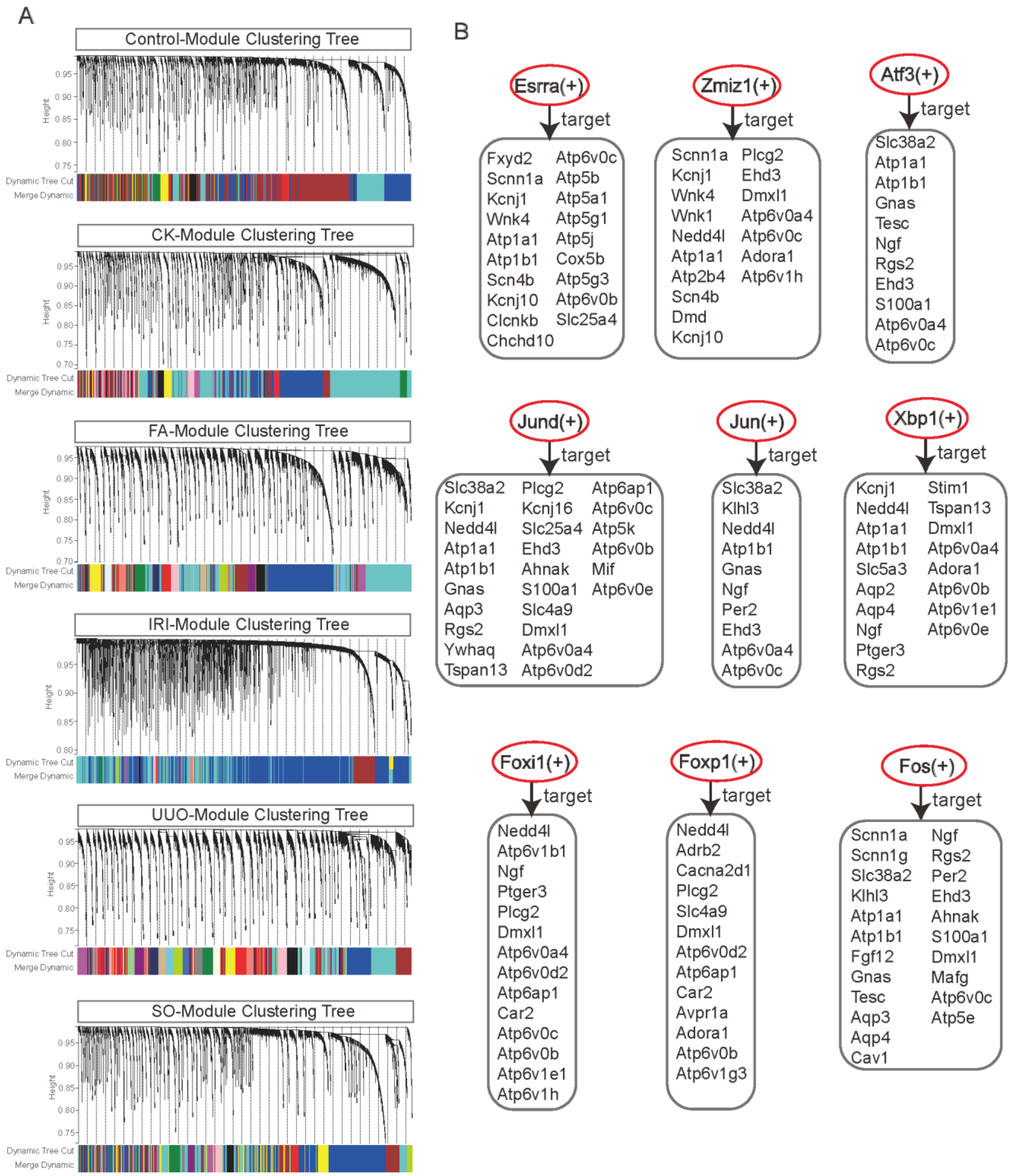

Supplemental Fig. S18. Module clustering tree of WGCNA for different groups and the regulatory pattern of key transcription factors.

(A) Control and 5 groups of AKI samples formed the topological overlap matrix (TOM) during the weighted gene co-expression network analysis (WGCNA). Genes in the TOM matrix were spontaneously formed into clusters based on expression patterns, and similar modules were merged by the dynamic shear tree method to finally generate the module clustering tree.

(B) Nine key transcription factors are involved in the cellular homeostasis of DCTs, LOH, CDIC and CDPC in AKI under stress conditions by regulating most genes of a series of basic biological processes (ion transfer, water homeostasis, pH regulation). Among them, *Atf3*, *Jun*, *Fos*, *Jund*, and *Zmiz1* are also involved in the regulation of apoptosis and autophagy pathways. In these four cell types, autophagy and apoptosis are the more common modes of injury.

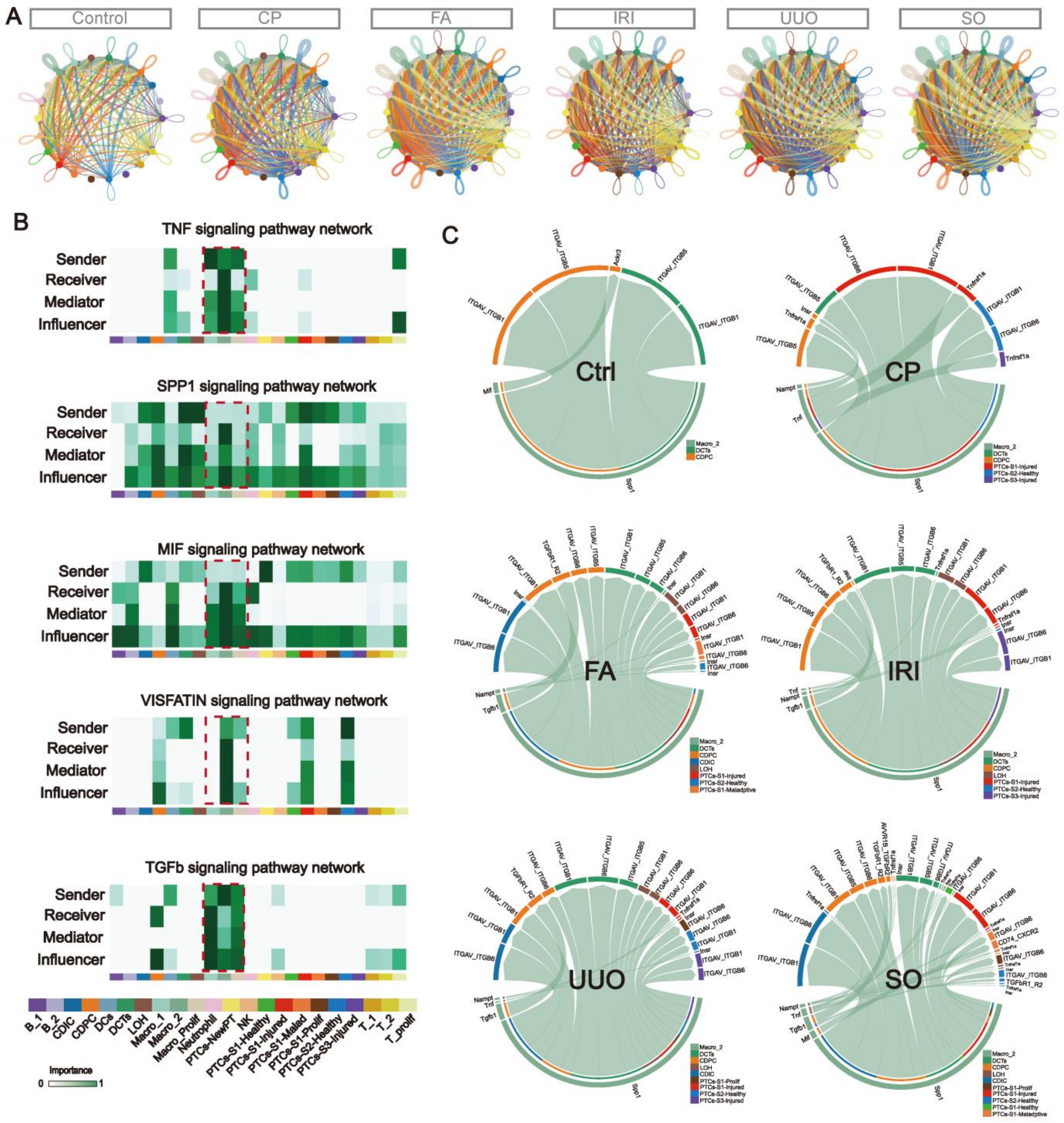

Supplemental Fig. S19. Model heterogeneity of signaling pathways corresponding to the significantly enriched ligand-receptors in Macro_2 cells.

(A) The overall cellular communication network map of Control and each AKI model, incorporated cells included 11 immune cell subtypes, 7 subtypes of PTCs, DCTs, LOH, CDIC, and CDPC. Compared with Control, the number and weight of intercellular communications markedly increased after the occurrence of AKI. Corresponding to **Figure 7B**. Share legend colors with panel B.

(B) The five signaling pathways corresponding to significantly enriched ligand-receptors (**Figure 7E**) are separately identified as the primary senders, receivers, mediators, and influencers in the AKI network by calculating the degree of information flow outside, inside, and in-between the communication network. It will help to assess the specific role of each cell type in the signaling pathways.

(C) The specific ligand-receptor signals of the five signaling pathways in Panel B were enriched in Control and each AKI model. The direction of Information flow is from Macro_2 cells to all parenchymal cells (PTC subtypes, DCTs, LOH, CDIC, and CDPC). Compared to Control, *Arg1*$^{+}$ Macro_2 cells targeted more parenchymal cells and activated more enriched intercellular ligand-receptor signaling in the AKI model.

Captions for Supplementary Tables S1 to S6

Supplemental Table S1: Differentially expressed genes in each cell cluster during initial cell clustering and cell re-clustering.

Supplemental Table S2: Differentially expressed genes in each celltypes.

Supplemental Table S3: Differentially expressed genes in each PTC sub-types.

Supplemental Table S4: Genes associated with cell differentiation in BEAM analysis of PTCs.

Supplemental Table S5: Differentially expressed genes in each ECs sub-types.

Supplemental Table S6: Key genes of DCTs, LOH, CDIC and CDPC in Control and each AKI model screened by WGCNA.
